# The salivary microbiota is altered in cervical dysplasia patients and influenced by conization

**DOI:** 10.1002/imt2.108

**Published:** 2023-05-12

**Authors:** Shengru Wu, Liqin Cheng, Alexandra A. L. Pennhag, Maike Seifert, Unnur Guðnadóttir, Lars Engstrand, Miriam Mints, Sonia Andersson, Juan Du

**Affiliations:** ^1^ Department of Microbiology, Tumor and Cell Biology, Centre for Translational Microbiome Research Karolinska Institute Stockholm Sweden; ^2^ College of Animal Science and Technology Northwest A&F University Yangling China; ^3^ Science for Life Laboratory Karolinska Institute Stockholm Sweden; ^4^ Department of Women's and Children's Health Karolinska Institute Stockholm Sweden

## Abstract

This study supports the correlation between the salivary microbiota and cervical dysplasia and suggests that smoking influences the salivary microbiota.

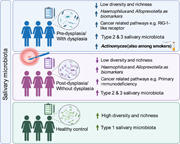

Cervical cancer is the fourth most commonly detected cancer in women worldwide [1]. The World Health Organization recently launched a global strategy to accelerate the elimination of cervical cancer [2, 3]. Despite the availability of vaccines for human papillomavirus (HPV) infection, which is the primary cause of cervical cancer, it is important to note that the current vaccines do not cover all oncogenic HPV types [4, 5, 6]. HPV testing and cytology testing (e.g., Pap smear) currently serve as the early screening methods for dysplasia and cervical cancer, after which histology can accurately identify the occurrence and stages of dysplasia. HPV testing and cytology testing have reported a significantly increased false rate compared with invasive histology diagnostic testing [7]. Hence, it is worth searching for a potential novel biomarker to improve the accuracy of early detection. Early detection, broader surveillance, and increased access to medical care and treatment would greatly assist cancer prevention, especially in low‐income countries [8].

Emerging evidence supports that vaginal microbiota correlate to HPV infection and dysplasia [9, 10, 11, 12]. However, data related to oral or salivary microbiota and cervical dysplasia are still limited. Whether oral microbiota play a role at different cervical dysplasia stages and how lifestyle influences salivary microbiota are important to evaluate. Poor oral hygiene and tooth loss are associated with an increased risk of oral squamous cell cancer [13]. Several specific oral bacterial pathogens, including *Fusobacterium*, *Campylobacter*, *Prevotella*, *Pseudomonas*, and *Capnocytophaga*, are correlated with lung, oral, esophageal, stomach, pancreatic, and colorectal cancers [14, 15, 16]. Notably, as saliva collection is noninvasive and the procedure is quicker, cheaper, and more convenient for the patient as compared to invasive processes such as blood collection, cytology, and histology testing. The salivary microbiota also serves as a biomarker for different cancers and systemic diseases diagnosis [14, 15, 16, 17, 18, 19].

Other known risk factors for cervical cancer include smoking, increased parity, and infection with the human immunodeficiency virus (HIV) [20]. Numerous studies have shown that smoking is a significant risk factor for developing cervical abnormalities, including cervical dysplasia and cervical cancer [21, 22]. Furthermore, lifestyles, such as diet, smoking, and alcohol intake, have been suggested to affect the oral microbiota and different diseases [23, 24, 25, 26, 27].

Herein, saliva samples were collected from both individuals undergoing cervical dysplasia examination and volunteers visiting the dental clinic for dental examinations. The study investigated the salivary microbiota and its potential relation to the occurrence of cervical dysplasia. Additionally, the study compared the salivary microbes that differed between volunteer controls and participants undergoing vaginal examination with and without dysplasia or pre‐ and postconization (Figure 1A). Furthermore, the lifestyle of participants through questionnaire data was investigated, and factors that may potentially influence oral microbiota were identified.

**Figure 1:**
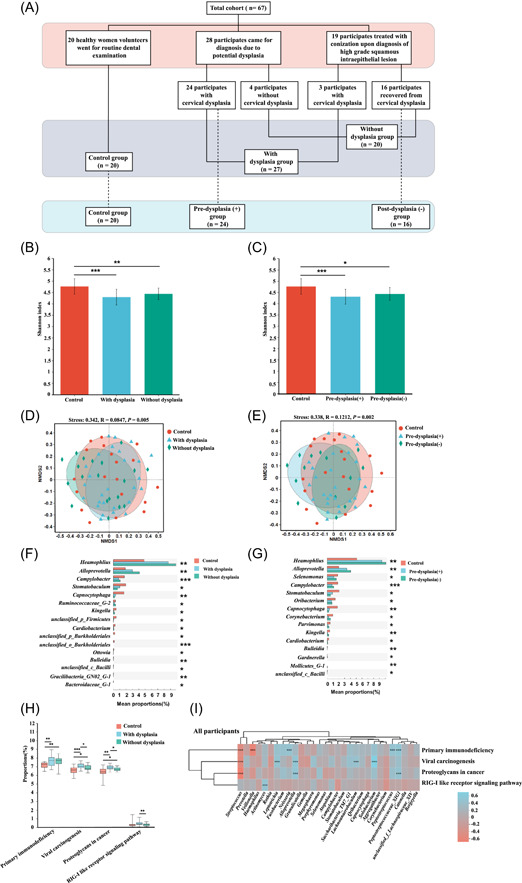
The study design and the comparison of salivary microbial alpha diversity, beta diversity, salivary genera, and bacterial functions of all the participants. (A) The study design and comparison groups. This cross‐sectional study recruited 20 healthy volunteers who visited for a routine dental examination at a dental clinic and 47 participants who visited for a vaginal examination at Karolinska University Hospital. Among these 47 women, 28 visited due to potential dysplasia, 19 of whom were treated with conization after being diagnosed with a high‐grade squamous intraepithelial lesion (HSIL). Histological data showed that 8 women had low‐grade squamous intraepithelial lesion (LSIL), 19 had HSIL, and 20 were within normal limits (WNL). We grouped individuals with WNL as a group without dysplasia and LSIL combined with HSIL as the dysplasia group. In addition, 24 of 28 participants, who came for checking potential dysplasia and confirmed with dysplasia, were identified as the predysplasia (+) group. The 16 of the 19 participants who visited for a follow‐up examination after conization treatment and confirmed dysplasia‐free were grouped into the postdysplasia (−) group. (B) The Shannon index was compared among participants with and without cervical dysplasia, as well as the participants who visited for the dental examination (control group). (C) The Shannon index was compared among patients from the predysplasia (+) and postdysplasia (−) groups and participants from the control group. The Kruskal–Wallis test and the Tukey–Kramer post hoc test were used to test microbial alpha diversity differences between more than two groups. ﻿﻿False discovery rate (FDR) < 0.05 **FDR < 0.01, ***FDR < 0.001. (D) The salivary microbial beta diversity (nonmetric multidimensional scaling [NMDS] analysis with amplicon sequence variants [ASVs] based on Bray–Curtis distance matrices and ANOSIM analysis) was compared among participants with and without cervical dysplasia and from the control group. (E) The salivary microbial beta diversity was compared among patients from the predysplasia (+) and postdysplasia (−) groups and participants from the control group. (F) The significantly changed salivary microbiota genera from the comparison among participants with and without cervical dysplasia and the control group. (G) The significantly changed salivary microbiota genera from the comparison among patients from the predysplasia (+) and postdysplasia (−) groups and participants from the control group. (H) The significantly changed salivary microbiota predicted immune‐related functions from the comparison among participants with and without cervical dysplasia and the control group. FDR < 0.05, **FDR < 0.01, ***FDR < 0.001. (I) The correlation between differential salivary bacterial functions and the 30 most abundant genera based on the data from all 67 participants. The differential salivary bacterial functions were identified from the comparison between participants with and without cervical dysplasia and the control group. The Kruskal–Wallis test with the Tukey–Kramer post hoc test was used to test for differences in microbial genera and function. For correlation analysis, only Spearman's rank correlation coefficient greater than 0.4 and a FDR less than 0.05 are presented. The blue grid indicates a positive correlation, and the red grid indicates a negative correlation. ***FDR < 0.001.

## RESULTS

### Differential salivary microbiota diversity was identified

Significantly decreased microbial community richness and microbial diversity were observed in participants with and without dysplasia when compared to the control group (Figure 1B, Supporting Information: Figure S1A). Moreover, significantly decreased microbial diversity was observed in participants of the predysplasia (+) and postdysplasia (−) groups and in participants from different histological groups compared to the control group (Figure 1C, Supporting Information: Figures S1B–D).

A significant dissimilarity was observed when we compared the control group with the groups with and without dysplasia (*P*_ANOSIM_ = 0.005) and with the predysplasia (+) and postdysplasia (−) (*P*_ANOSIM_ = 0.002) (Figure 1D,E). However, no significant differences were identified among the control and different histological groups (Supporting Information: Figure S1E). All oral samples from vaginal examination participants tested HPV negative. The PERMANOVA analysis considered the overall effect of all participants' lifestyles on the microbiota beta diversity and found that age and smoking could influence microbiota diversity (Supporting Information: Tables S1 and S2, Supporting Information: Figure S2).

### Certain salivary bacteria assisted in distinguishing the patients with cervical dysplasia from healthy participants

The top five main genera and species found belong to *Streptococcus*, *Prevotella*, *Veillonella*, *Haemophilus*, and *Actinomyces* (Supporting Information: Figures S3 and S4). When comparing the salivary microbiota of participants with and without dysplasia with healthy participants, up to 16 different genera were identified as having significantly different relative abundance (Figure 1F). A similar trend was observed when comparing groups of predysplasia (+) and postdysplasia (−). The relative abundance of *Haemophlius* and *Alloprevotella* was significantly increased among saliva samples from vaginal examination participants than those from the healthy participants (Figure 1G).

Based on the reconstruction of unobserved states 2 (PICRUSt2) analysis, the pathways of primary immunodeficiency, viral carcinogenesis, proteoglycans in cancer, and retinoic acid‐inducible gene I (RIG‐I)‐like receptor signaling pathways were significantly changed when comparing the salivary microbiota predicted function of participants with and without dysplasia with healthy participants (Figure 1H), as well as comparing the pre‐ysplasia (+) and postdysplasia (−) participants with healthy participants. The high‐relative‐abundance bacterial genera, such as *Streptococcus* and *Veillonella*, were negatively correlated to the pathways of primary immunodeficiency, viral carcinogenesis, and proteoglycans in cancer. While other genera, such as *Alloprevotella*, were positively correlated with the viral carcinogenesis and proteoglycans in cancer, and *Actinomyces* was positively correlated with RIG‐I‐like receptor (RLR) signaling pathways (Figure 1I). Furthermore, when comparing the control group with the group with or without dysplasia, *Prevotella, Haemophilus and Alloprevotella* were found to be significantly increased in the latter groups compared to the control group (Supporting Information: Figure S5A–B). Moreover, *Haemophilus* and *Alloprevotella* were the genera significantly higher among participants from the predysplasia (+) and postdysplasia (−) groups than the control group (Supporting Information: Figure S5C–D). The area under the curve (AUC) of *Haemophlius* (*p* = 0.0039), *Alloprevotella* (*p* = 0.0076), and *Prevotella* (*p* = 0.0350) reached 0.748, 0.730, and 0.682, respectively, which indicates that these three genera could support the diagnosis of patients with dysplasia from healthy condition (Supporting Information: Figure S5E–G).

### Three salivary microbial types were identified

**Figure 2:**
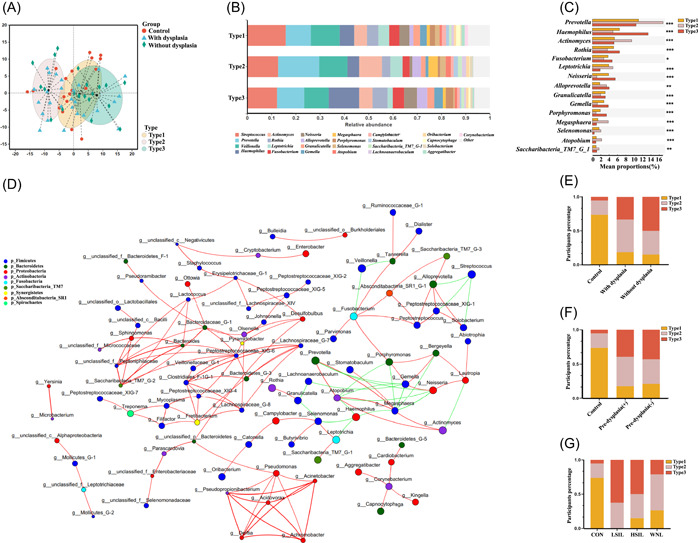
Salivary microbial types of all the participants. (A) Three different salivary microbial types were identified based on the genera of salivary microbes. The partitioning around medoids (PAM) cluster based on the Jensen–Shannon distance (JSD) and the Calinski–Harabasz (CH) indices was used. (B) The comparison of microbial composition among three different salivary microbial types at the genus level. (C) The significantly altered genera among three different salivary microbial types. The Kruskal–Wallis test with the Tukey–Kramer post hoc test was used to test differences in the microbial genera. *FDR < 0.05, **FDR < 0.01, ***FDR < 0.001. (D) The correlation network among genera in salivary microbiota based on Spearman's rank correlation coefficient analysis. Only Spearman's rank correlation coefficient greater than 0.5 and a FDR less than 0.05 are presented. The red line represents a positive correlation, and the green line represents a negative correlation. (E) The distribution of different microbiota types among participants with and without cervical dysplasia and the control group. (F) The distribution of different microbiota types among patients from the predysplasia (+) and postdysplasia (–) groups and the control group. (G) The distribution of different microbiota types among participants from the control group and the LSIL, HSIL, and WNL groups.

Based on the similarities in the genera identified from different samples, three different salivary microbial types were generated (Figure 2A). Type 2 contained a higher abundance of *Prevotella* and *Actinomyces*, which closely interacted according to our network analysis. Type 3 contained a higher abundance of *Haemophilus*, and Type 1 microbiota type contained a similar abundance of all the major genera (Figure 2B–D). Participants in the groups with and without dysplasia had higher percentages of type 2 and type 3 than the participants in the control group (Figure 2E). A similar trend was observed when comparing predysplasia (+) and postdysplasia (−) groups with the control group (Figure 2F) and when comparing low‐grade squamous intraepithelial lesion (LSIL) and high‐grade squamous intraepithelial lesion (HSIL) groups with the control group (Figure 2G). Moreover, a tightly interactive network was formed by *Prevotella*, *Actinomyces*, and *Haemophilus*, indicating a potential relationship between salivary microbiota and their distinct contributions to various diseases and treatment outcomes (Figure 2D).

### Salivary *Actinomyces* assisted in distinguishing patients with and without cervical dysplasia

**Figure 3:**
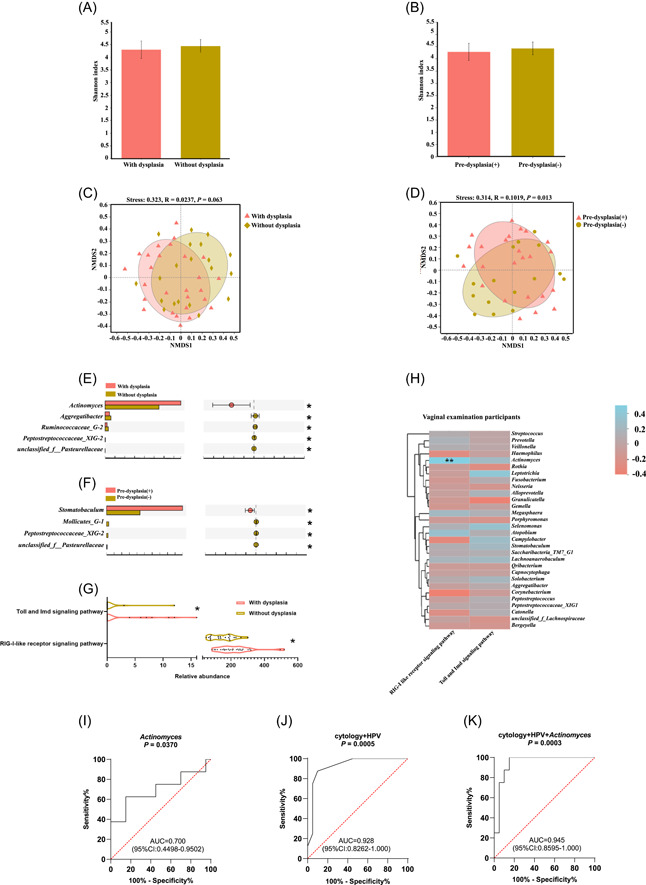
Comparison of salivary microbial alpha diversity, beta diversity, salivary genera, and salivary bacterial functions between participants with and without cervical dysplasia and between participants from the predysplasia (+) and postdysplasia (−) groups. (A) The Shannon index was compared between the participants with and without cervical dysplasia. (B) The Shannon index was compared between the patients from the predysplasia (+) and postdysplasia (−) groups. The Mann–Whitney U test was carried out to compare the two groups. (C) The salivary microbial beta diversity was compared between the participants with and without cervical dysplasia. (D) The salivary microbial beta diversity was compared between the patients from the predysplasia (+) and postdysplasia (−) groups. (E) The significantly changed salivary microbiota genera from the comparison between participants with and without cervical dysplasia. *FDR < 0.05. (F) The significantly changed salivary microbiota genera from the comparison between patients from the predysplasia (+) and postdysplasia (−) groups. *FDR < 0.05. (G) The cosignificantly changed salivary microbiota predicted immune‐related functions from the comparison between patients with and without cervical dysplasia. *FDR < 0.05. (H) The correlation between the codifferential salivary bacterial functions and the 30 most abundant genera based on the data from the 47 vaginal examination participants. The differential salivary bacterial functions were identified from the comparison among participants with and without cervical dysplasia. The Mann–Whitney U test was carried out to compare the two groups. For correlation analysis, only Spearman's rank correlation coefficient greater than 0.4 and a false discovery rate (FDR) less than 0.05 are presented. The blue grid indicates a positive correlation, and the red grid indicates a negative correlation. **FDR < 0.01. (I–K) The area under the curve (AUC) of receiver operating characteristic (ROC) analyses helps to identify the diagnostic accuracy of participants with and without cervical dysplasia based on the salivary *Actinomyces* abundance (I), the conventional vaginal cytology and HR‐HPV testing (J), as well as the conventional vaginal cytology and HR‐HPV testing plus salivary *Actinomyces* abundance (K).

All the comparisons were further evaluated without the dental examination control group (Figure 3A–D and Supporting Information: Figure S6). PERMANOVA analysis was also carried out to consider the overall effect of the participants' lifestyles for salivary microbial composition (Supporting Information: Tables S3 and S4). Only microbiota beta diversity between the predysplasia (+) and postdysplasia (−) participants was significantly altered (Figure 3D). In addition, *Actinomyces* was significantly increased in the dysplasia group, and bacteria, including *Aggregatibacter*, was significantly increased in the group without dysplasia (Figure 3E). Further, in the comparison of the predysplasia (+) and postdysplasia (−) participants, *Stomatobaculum* was significantly increased among the predysplasia (+) participants (Figure 3F).

*Actinomyces* was found to be correlated with increased RLR signaling, which is elevated in patients with dysplasia (Figures 3G,H). The diagnostic ability of oral *Actinomyces* abundance alone reached 0.700 (*p* = 0.0370), which indicates that *Actinomyces* could help to diagnose dysplasia patients from the ones without dysplasia (Figure 3I). Notably, combining cytology and HPV infection testing with the oral *Actinomyces* abundance, the diagnostic ability improved to the classification of AUC = 0.945 (*p* = 0.0003) compared to conventional cytology combined with HPV infection testing (AUC = 0.928, *p* = 0.0005) (Figure 3J–K).

### *Actinomyces* had a higher abundance among smokers than nonsmokers

Since smoking significantly influenced the salivary microbial beta diversity, as revealed by the PERMANOVA analysis (Supporting Information: Tables S1 and S2), the differences in salivary microbiota alpha and beta diversity that were affected by smoking (both previous and current smokers) were further investigated (Supporting Information: Figure S7). No significant alpha diversity was observed between smoker and nonsmoking groups (Supporting Information: Figure S7A–D). However, the smokers had significantly altered salivary microbiota beta diversity when compared with participants who never smoked (Supporting Information: Figure S7E,F). Furthermore, the salivary microbial types distribution among smokers and nonsmokers was also analyzed. The smokers had higher percentages of type 2 (containing a higher abundance of *Prevotella* and *Actinomyces*) than the nonsmokers (Supporting Information: Figure S7G). Interestingly, when we investigated the bacteria that were significantly altered between smokers and nonsmokers, *Actinomyces* was once again significantly increased among smokers compared to nonsmokers (Supporting Information: Figure S7H,I).

## DISCUSSION

Our study found that participants who visited the hospital for a vaginal dysplasia examination had significantly decreased oral microbiota richness and diversity compared to control participants who visited for a dental examination. This is in line with the earlier studies that link a decreased oral microbiota diversity to the occurrence of different kinds of cancers, such as throat cancer, lung cancer, and head and neck cancer [28, 29, 30, 31]. We also found that the salivary microbiota diversity of patients who had recovered from cervical dysplasia after conization differed from that of patients with cervical dysplasia before treatment. Notably, significantly distinguished microbiota beta diversities were identified among the control group, the groups with and without dysplasia, and the groups predysplasia (+) and postdysplasia (−).

By using the eHOMD database, *Streptococcus*, *Prevotella*, *Veillonella*, and *Haemophilus* were among the most abundant genera, and *Haemophilus parainfluenzae, Veillonella atypica*, and *Rothia mucilaginosa* were among the most abundant species in the present study, which is in line with previous publications [32, 33, 34]. Furthermore, three different salivary microbial types were identified, and both the percentage of *Prevotella*‐enriched and *Haemophilus*‐enriched types were increased in participants with and without dysplasia compared with the control group. *Actinomyces*, the key abundant genera in type 2, which had a positive correlation with *Prevotella*, showed a significantly higher abundance in patients with dysplasia than the ones without dysplasia. Notably, the increase of *Prevotella*, *Haemophilus*, and *Alloprevotella* in the saliva of current or past dysplasia patients could serve as a biomarker to distinguish them from the healthy participants. Furthermore, the increase of *Actinomyces* could serve as a biomarker for distinguishing the current dysplasia patients from past dysplasia patients. Similar to the diagnostic roles of salivary microbiota in other kinds of cancers, [14, 15, 16, 17, 18, 19] our results expanded the use of the salivary microbiota as the noninvasive diagnostic biomarker for cervical dysplasia.

Elevated levels of oral *Prevotella*, *Haemophilus*, and *Alloprevotella* have been linked to various oral diseases, including periodontal disease, tonsillar disease, and oral cavity squamous cell carcinoma [35, 36]. These same microbial types have also been associated with systemic diseases, such as gout, obesity, cardiovascular disease, and liver cancer [37, 38, 39, 40, 41]. Furthermore, increased levels of *Actinomyces* have been linked to suppurative, granulomatous inflammatory lesions and have been shown to induce cervicofacial, pulmonary, and abdominopelvic infections, as well as oral cancer [42, 43, 44, 45]. Other studies imply that these four genera are all oral opportunistic pathogens and can be linked to inflammatory reactions and cancer development [16, 39, 46, 47, 48, 49, 50, 51, 52, 53, 54, 55, 56, 57]. Notably, a significant increase of *Prevotella* and *Actinomyces* has been previously reported in vaginal or cervical exudates from women with cervical dysplasia or cervical cancer [10, 58, 59]. This finding suggests the potential for the transfer of these opportunistic pathogens between the oral and vaginal tracts.

Oral sexual behavior is one of the main routes for the transfer of bacteria or viruses from the oral to the vaginal tract. Previous studies have shown that most oral HPV types match the co‐occurring cervical HPV types, indicating the potential for transfer between these two sites [60]. Furthermore, oral intake of *Lactobacillus* probiotics has been shown to increase their relative abundance in the vagina [61]. In addition, metabolites or immune cells induced by oral microorganisms or virulent strains may enter the systemic circulation through the blood, leading to low‐grade inflammation and promoting the development of chronic inflammatory diseases and cancer in organs beyond the oral tract [62, 63]. We identified potential microbial roles in differential microbial immune‐related pathways, revealing that Toll and Imd signaling pathways, as well as RLR, showed increased activity in the dysplasia and pretreatment groups. This indicates a potential increase in oral inflammation [64]. Previous studies have indicated that increased oral inflammation can activate Th1 or Th17 cells, leading to bacteremia or systemic inflammation [17, 63, 65, 66].

Our study indicates that smoking can affect the composition of salivary microbiota, which is supported by previous studies [67, 68, 69, 70]. *Actinomyces*, which is significantly increased among smokers in our study, had been linked to higher gingivitis risk among cigarette smokers in other studies [67, 68, 69, 70, 71]. Cigarette smoking is an independent risk factor for cervical dysplasia occurrence and development [21, 59, 72, 73]. We identified a significant positive relationship between the RLR pathway and *Actinomyces* among patients with dysplasia than the ones without, and among smokers than nonsmokers. This supports a potential role for oral microbiota in linking smoking, oral inflammation, and dysplasia development. Further mechanistic and longitudinal studies that include smokers are needed to track the complicated interactions and potential talk between oral microbiota and cervical cancer development.

There are several limitations to the present study. First, the limited number of patients and the complex situation of the recruited participants may have affected the oral microbiota data and the identification of potential diagnostic bacterial biomarkers, which must be considered when interpreting the results. In addition, smoking, age distribution, and other living habits, such as the use of antibiotics, may affect the identified microbes [74]. However, the PERMANOVA analysis suggests a significant difference in microbiota between the participants with and without cervical dysplasia, as well as between predysplasia (+) and postdysplasia (−) groups considering age and other living habits as confounders. These results indicated that differential genera, such as *Actinomyces* and *Stomatobaculum*, may contribute to the occurrence and recovery of vaginal dysplasia. Moreover, the detailed oral conditions of the participants who underwent vaginal examination, such as the periodontal disease or the decayed, missing, and filled (DMF) index, were not well identified, which may also affect the oral microbiota [75, 76]. Further, the dental examination itself may also have potential selection biases for the result. Thus, the data without the dental examination group were presented. It is worth noting that the taxonomic classification at the species level using 16S rRNA gene sequencing may not be entirely reliable, and the use of PICRUSt2 to predict microbial function may not fully reflect the actual metagenome changes. However, using PICRUSt2 could provide novel insights into microbial functions that help to search for potential biomarkers [77]. Moreover, given that those four identified potential biomarker genera, including the *Prevotella*, *Alloprevotella*, *Haemophilus*, and *Actinomyces*, are all opportunistic pathogens in the oral cavity and can be linked to inflammatory reactions and cancer development, we believe that our results provide valuable information to identify additional potential salivary targets for smoking‐related diseases and cervical dysplasia. Last, most of the participants without dysplasia previously experienced dysplasia, which might affect the representativeness of their data as a healthy state reference. However, our study provides one of the first insights into the association between salivary microbiota and cervical dysplasia and its treatment. Given that certain bacteria were identified in multiple comparisons, our study lays a valuable foundation for further research on the role of salivary microbiota in cervical cancer. Additional longitudinal studies focusing on the metagenome changes in the oral and vaginal tracts during disease development and recovery are urgently needed to gain a deeper understanding of the involvement of salivary microbiota.

## CONCLUSION

This study demonstrated that salivary microbiota profiles differed between participants with and without cervical dysplasia, as well as between patients before and after cervical conization. Moreover, the study suggests that *Actinomyces* in the salivary microbiota may link smoking, oral microbiota, and cervical dysplasia and could potentially serve as a diagnostic marker.

## MATERIALS AND METHODS

### Study design

This cross‐sectional study involved 47 women who visited the Karolinska University Hospital in Stockholm, Sweden. In this study, 28 women visited due to potential dysplasia and 19 visited for follow‐up examination after treatment with conization for HSIL (Figure 1A and Supporting Information: Table S5). Furthermore, 20 healthy women who volunteered for a routine dental examination at a dental clinic in Stockholm, Sweden, were also included in the study. All participants were asked to fill out a questionnaire regarding their lifestyles (Supporting Information: Table S6). The study was approved by the Regional Ethical Board in Stockholm, Sweden. All participants provided written informed consent to take part in the study. Detailed diagnostic and sample collection methods are available in the Supplementary material.

### Oral microbiota sequencing

DNA was extracted from all 67 saliva samples and used for HPV genotyping with the MAGPIX instrument, according to our published papers [6, 12, 78, 79]. Furthermore, the V3–V4 regions of the 16S rRNA genes were amplified using Illumina sequencing index‐binding primer pairs 341F/805 R and sequenced on an Illumina MiSeq sequencing platform [37]. Thereafter, the sequencing data were analyzed using the QIIME2 platform and the Human Oral Microbiome Database (eHOMD) (V15.2). Detailed information about DNA extraction, oral HPV genotyping, oral microbiota sequencing, and bioinformatic analyses is available in the Supplementary material.

### Statistics

The Mann–Whitney U test with multiple comparisons adjusted with the Benjamini–Hochberg FDR was performed to compare microbial alpha diversity and identify significantly altered genera and KEGG pathways between two groups. The Kruskal–Wallis test with the Tukey–Kramer post hoc test was used to test microbial differences among more than two groups. ANOSIM analysis based on Bray–Curtis distance matrices was used to identify beta diversity, and the adonis function from the R package “vegan” was used for PERMANOVA [80, 81]. The pairwise correlations (*p* < 0.05) were used to generate the co‐occurrence network (Spearman's rank correlation coefficient indices > 0.5) and correlation heatmaps (Spearman's rank correlation coefficient indices > 0.4). Additionally, the AUC of the receiver operating characteristic was calculated to analyze the sensitivity and specificity of the diagnostic power.

## AUTHOR CONTRIBUTIONS

*Conception and design*: Shengru Wu, Lars Engstrand, Sonia Andersson, Juan Du. *Sample collection*: Liqin Cheng, Alexandra A. L. Pennhag, Miriam Mints, Sonia Andersson, Juan Du. *Development of methodology*: Shengru Wu, Liqin Cheng, Alexandra A. L. Pennhag, Maike Seifert, Juan Du. *Acquisition of data*: Shengru Wu, Liqin Cheng, Alexandra A. L. Pennhag, Maike Seifert, Unnur Guðnadóttir, Miriam Mints, Sonia Andersson, Juan Du. *Analysis and interpretation of data*: Shengru Wu, Liqin Cheng, Sonia Andersson, Juan Du. *Writing of the manuscript*: Shengru Wu, Juan Du. *Review and/or revision of the manuscript*: All authors.

## CONFLICT OF INTEREST STATEMENT

The authors declare no conflict of interest.

## ETHICS STATEMENT

The study was approved by the Regional Ethical Board at Karolinska Institute, Stockholm, Sweden (ethical permission number 2017/725‐31 and 2019‐04201).

## Supporting information

Supporting information.

Supporting information.

## Data Availability

All the data generated from this study are included in this paper. The sequencing reads are available in the Sequence Read Archive (SRA) of NCBI under accession project number PRJNA863336 (https://www.ncbi.nlm.nih.gov/bioproject/PRJNA863336). Supplementary materials (figures, tables, scripts, graphical abstract, slides, videos, Chinese translated version, and updated materials) can be found in the online DOI or iMeta Science http://www.imeta.science/.

## References

[1] Arbyn, Marc , Elisabete Weiderpass , Laia Bruni , Silvia de Sanjosé , Mona Saraiya , Jacques Ferlay , and Freddie Bray . 2020. “Estimates of Incidence and Mortality of Cervical Cancer in 2018: A Worldwide Analysis.” The Lancet Global Health 8: e191–203. 10.1016/S2214-109X(19)30482-6 31812369 PMC7025157

[2] Gultekin, Murat , Pedro T. Ramirez , Nathalie Broutet , and Raymond Hutubessy . 2020. “World Health Organization Call for Action to Eliminate Cervical Cancer Globally.” International Journal of Gynecologic Cancer 30: 426–7. 10.1136/ijgc-2020-001285 32122950

[3] Allanson, Emma R. , and Kathleen M. Schmeler . 2021. “Preventing Cervical Cancer Globally: Are We Making Progress?” Cancer Prevention Research 14: 1055–60. 10.1158/1940-6207.CAPR-21-0016 34853026

[4] Cheng, Liqin , Yan Wang , and Juan Du . 2020. “Human Papillomavirus Vaccines: An Updated Review.” Vaccines 8: 391. 10.3390/vaccines8030391 32708759 PMC7565290

[5] Canfell, Karen , Jane J. Kim , Marc Brisson , Adam Keane , Kate T. Simms , Michael Caruana , Emily A. Burger , et al. 2020. “Mortality Impact Of Achieving WHO Cervical Cancer Elimination Targets: a Comparative Modelling Analysis in 78 Low‐Income and Lower‐Middle‐Income Countries.” The Lancet 395: 591–603. 10.1016/S0140-6736(20)30157-4 PMC7043006 32007142

[6] Ährlund‐Richter, Andreas , Liqin Cheng , Yue O. O. Hu , Mikaela Svensson , Alexandra A. L. Pennhag , Ramona G. Ursu , Linnea Haeggblom , et al. 2019. “Changes in Cervical Human Papillomavirus (HPV) Prevalence at a Youth Clinic in Stockholm, Sweden, a Decade After the Introduction of the HPV Vaccine.” Frontiers in Cellular and Infection Microbiology 9: 59. 10.3389/fcimb.2019.00059 30949454 PMC6435486

[7] Zheng, Baowen , Zaibo Li , Christopher C. Griffith , Shanshan Yan , Congde Chen , Xiangdong Ding , Xiaoman Liang , Huaitao Yang , and Chengquan Zhao . 2015. “Prior High‐Risk HPV Testing and Pap Test Results for 427 Invasive Cervical Cancers in China's Largest CAP‐certified Laboratory: Prior High‐Risk HPV Testing.” Cancer Cytopathology 123: 428–34. 10.1002/cncy.21557 25954852

[8] Khan, Michelle J. , and Karen K. Smith‐McCune . 2014. “Treatment of Cervical Precancers: Back to Basics.” Obstetrics Gynecology 123: 1339–43. 10.1097/AOG.0000000000000287 24807323 PMC4077778

[9] Kawahara, Rina , Takuma Fujii , Iwao Kukimoto , Hiroyuki Nomura , Rie Kawasaki , Eiji Nishio , Ryoko Ichikawa , Tetsuya Tsukamoto , and Aya Iwata . 2021. “Changes to the Cervicovaginal Microbiota and Cervical Cytokine Profile Following Surgery for Cervical Intraepithelial Neoplasia.” Scientific Reports 11: 2156. 10.1038/s41598-020-80176-6 33495564 PMC7835242

[10] Mitra, Anita , David A. MacIntyre , George Ntritsos , Ann Smith , Konstantinos K. Tsilidis , Julian R. Marchesi , Phillip R. Bennett , Anna‐Barbara Moscicki , and Maria Kyrgiou . 2020. “The Vaginal Microbiota Associates With the Regression of Untreated Cervical Intraepithelial Neoplasia 2 Lesions.” Nature Communications 11: 1999. 10.1038/s41467-020-15856-y PMC7181700 32332850

[11] Wu, Mengying , Jing Gao , Yongqin Wu , Yanyun Li , Yisheng Chen , Fuju Zhao , Cui Li , et al. 2020. “Characterization of Vaginal Microbiota in Chinese Women with Cervical Squamous Intra‐Epithelial Neoplasia.” International Journal of Gynecological Cancer 10: 30. 10.1136/ijgc-2020-001341 32499394

[12] Cheng, Liqin , Johanna Norenhag , Yue O. O. Hu , Nele Brusselaers , Emma Fransson , Andreas Ährlund‐Richter , Unnur Guðnadóttir , et al. 2020. “Vaginal Microbiota and Human Papillomavirus Infection Among Young Swedish Women.” Npj Biofilms and Microbiomes 6: 39. 10.1038/s41522-020-00146-8 33046723 PMC7552401

[13] Yao, Qi‐Wei , Dong‐Sheng Zhou , Hong‐Juan Peng , Ping Ji , and De‐Sheng Liu . 2014. “Association of Periodontal Disease With Oral Cancer: A Meta‐Analysis.” Tumor Biology 35: 7073–7. 10.1007/s13277-014-1951-8 24756759

[14] Flemer, Burkhardt , Ryan D. Warren , Maurice P. Barrett , Katryna Cisek , Anubhav Das , Ian B. Jeffery , Eimear Hurley , et al. 2018. “The Oral Microbiota in Colorectal Cancer is Distinctive and Predictive.” Gut 67: 1454–63. 10.1136/gutjnl-2017-314814 28988196 PMC6204958

[15] Farrell, James J. , Lei Zhang , Hui Zhou , David Chia , David Elashoff , David Akin , Bruce J. Paster , Kaumudi Joshipura , and David T. W. Wong . 2012. “Variations of Oral Microbiota Are Associated with Pancreatic Diseases Including Pancreatic Cancer.” Gut 61: 582–8. 10.1136/gutjnl-2011-300784 21994333 PMC3705763

[16] Fan, Xiaozhou , Alexander V. Alekseyenko , Jing Wu , Brandilyn A. Peters , Eric J. Jacobs , Susan M. Gapstur , et al. 2018. “Human Oral Microbiome and Prospective Risk for Pancreatic Cancer: A Population‐Based Nested Case‐Control Study.” Gut 67: 120–7. 10.1136/gutjnl-2016-312580 27742762 PMC5607064

[17] Gao, Lu , Tiansong Xu , Gang Huang , Song Jiang , Yan Gu , and Feng Chen . 2018. “Oral Microbiomes: More and More Importance in Oral Cavity and Whole Body.” Protein Cell 9: 488–500. 10.1007/s13238-018-0548-1 29736705 PMC5960472

[18] Peng, Xian , Lei Cheng , Yong You , Chengwei Tang , Biao Ren , Yuqing Li , Xin Xu , and Xuedong Zhou . 2022. “Oral Microbiota in Human Systematic Diseases.” International Journal of Oral Science 14: 14. 10.1038/s41368-022-00163-7 35236828 PMC8891310

[19] Wang, Xiaoqian , Karolina Elżbieta Kaczor‐Urbanowicz , and David T. W. Wong . 2017. “Salivary Biomarkers in Cancer Detection.” Medical Oncology 34: 7. 10.1007/s12032-016-0863-4 27943101 PMC5534214

[20] Stelzle, Dominik , Luana F. Tanaka , Kuan Ken Lee , Ahmadaye Ibrahim Khalil , Iacopo Baussano , Anoop S. V. Shah , David A. McAllister , et al. 2021. “Estimates of the Global Burden of Cervical Cancer Associated with HIV.” The Lancet Global Health 9: e161–9. 10.1016/S2214-109X(20)30459-9 33212031 PMC7815633

[21] Nagelhout, Gera , Renée Mf Ebisch , Olga Van Der Hel , Gert‐Jan Meerkerk , Tessa Magnée , Thomas De Bruijn , and Barbara Van Straaten . 2021. “Is Smoking an Independent Risk Factor for Developing Cervical Intra‐Epithelial Neoplasia and Cervical Cancer? A Systematic Review and Meta‐Analysis.” Expert Review of Anticancer Therapy 21: 781–94. 10.1080/14737140.2021.1888719 33663309

[22] Bandi, Priti , Adair K. Minihan , Rebecca L. Siegel , Farhad Islami , Nigar Nargis , Ahmedin Jemal , and Stacey A. Fedewa . 2021. “Updated Review of Major Cancer Risk Factors and Screening Test Use in the United States in 2018 and 2019, with a Focus on Smoking Cessation.” Cancer Epidemiology, Biomarkers Prevention 30: 1287–99. 10.1158/1055-9965.EPI-20-1754 34011554

[23] Jia, Yi‐Jing , Ying Liao , Yong‐Qiao He , Mei‐Qi Zheng , Xia‐Ting Tong , Wen‐Qiong Xue , Jiang‐Bo Zhang , et al. 2021. “Association Between Oral Microbiota and Cigarette Smoking in the Chinese Population.” Frontiers in Cellular and Infection Microbiology 11: 658203. 10.3389/fcimb.2021.658203 34123872 PMC8195269

[24] Zapała, Barbara , Tomasz Stefura , Tomasz Milewicz , Julia Wątor , Monika Piwowar , Magdalena Wójcik‐Pędziwiatr , Magdalena Doręgowska , et al. 2022. “The Role of the Western Diet and Oral Microbiota in Parkinson's Disease.” Nutrients 14: 355. 10.3390/nu14020355 35057536 PMC8778357

[25] Agarwal, Dhiraj M. , Dhiraj P. Dhotre , Shreyas V. Kumbhare , Akshay H. Gaike , Bill B. Brashier , Yogesh S. Shouche , Sanjay K. Juvekar , and Sundeep S. Salvi . 2021. “Disruptions in Oral and Nasal Microbiota in Biomass and Tobacco Smoke Associated Chronic Obstructive Pulmonary Disease.” Archives of Microbiology 203: 2087–99. 10.1007/s00203-020-02155-9 33598807

[26] Yuwanati, Monal , Sachin C. Sarode , Amol Gadbail , Shailesh Gondivkar , and Gargi S. Sarode . 2021. “Alcohol Attributed Oral Cancer and Oral Microbiome: Emerging yet Neglected Research Domain.” Oral Oncology 123: 105596. 10.1016/j.oraloncology.2021.105596 34715451

[27] Bostanci, Nagihan , Maria Christine Krog , Luisa W. Hugerth , Zahra Bashir , Emma Fransson , Fredrik Boulund , Georgios N. Belibasakis , et al. 2021. “Dysbiosis of the Human Oral Microbiome During the Menstrual Cycle and Vulnerability to the External Exposures of Smoking and Dietary Sugar.” Frontiers in Cellular and Infection Microbiology 11: 625229. 10.3389/fcimb.2021.625229 33816334 PMC8018275

[28] Wang, Lili , Gaofei Yin , Ying Guo , Yaqi Zhao , Meng Zhao , Yunyun Lai , Pengcheng Sui , et al. 2019. “Variations in Oral Microbiota Composition Are Associated With a Risk of Throat Cancer.” Frontiers in Cellular and Infection Microbiology 9: 205. 10.3389/fcimb.2019.00205 31334130 PMC6618584

[29] Yang, Junjie , Xiaofeng Mu , Ye Wang , Dequan Zhu , Jiaming Zhang , Cheng Liang , Bin Chen , et al. 2018. “Dysbiosis of the Salivary Microbiome Is Associated With Non‐Smoking Female Lung Cancer and Correlated With Immunocytochemistry Markers.” Frontiers in Oncology 8: 520. 10.3389/fonc.2018.00520 30524957 PMC6256243

[30] Sharma, Ashok Kumar , William T. DeBusk , Irina Stepanov , Andres Gomez , and Samir S. Khariwala . 2020. “Oral Microbiome Profiling in Smokers with and without Head and Neck Cancer Reveals Variations Between Health and Disease.” Cancer Prevention Research 13: 463–74. 10.1158/1940-6207.CAPR-19-0459 32071121 PMC13276710

[31] Hosgood, H Dean , Qiuyin Cai , Xing Hua , Jirong Long , Jianxin Shi , Yunhu Wan , Yaohua Yang , et al. 2021. “Variation in Oral Microbiome is Associated with Future Risk of Lung Cancer Among Never‐Smokers.” Thorax 76: 256–63. 10.1136/thoraxjnl-2020-215542 33318237 PMC8513501

[32] Sembler‐Møller, Maria Lynn , Daniel Belstrøm , Henning Locht , Christian Enevold , and Anne Marie Lynge Pedersen . 2019. “Next‐Generation Sequencing of Whole Saliva From Patients With Primary Sjögren's Syndrome and Non‐Sjögren's Sicca Reveals Comparable Salivary Microbiota.” Journal of Oral Microbiology 11: 1660566. 10.1080/20002297.2019.1660566 31497258 PMC6720018

[33] Belstrøm, Daniel , Josefine Maria Eiberg , Christian Enevold , Maria Anastasia Grande , Claus Antonio Juel Jensen , Lone Skov , and Peter Riis Hansen . 2020. “Salivary Microbiota and Inflammation‐Related Proteins in Patients with Psoriasis.” Oral Diseases 26: 677–87. 10.1111/odi.13277 31916654 PMC7188313

[34] Belstrøm, Daniel , Florentin Constancias , Yang Liu , Liang Yang , Daniela I. Drautz‐Moses , Stephan C. Schuster , Gurjeet Singh Kohli , et al. 2017. “Metagenomic and Metatranscriptomic Analysis of Saliva Reveals Disease‐Associated Microbiota in Patients with Periodontitis and Dental Caries.” Npj Biofilms and Microbiomes 3(1): 23. 10.1038/s41522-017-0031-4 28979798 PMC5624903

[35] Arredondo, Alexandre , Vanessa Blanc , Carolina Mor , José Nart , and Rubén León . 2019. “Azithromycin and Erythromycin Susceptibility and Macrolide Resistance Genes in Prevotella from Patients with Periodontal Disease.” Oral Diseases 25: 860–7. 10.1111/odi.13043 30667163

[36] Chen, Casey , Chris Hemme , Joan Beleno , Zhou Jason Shi , Daliang Ning , Yujia Qin , Qichao Tu , et al. 2018. “Oral Microbiota Of Periodontal Health and Disease and Their Changes After Nonsurgical Periodontal Therapy.” The ISME Journal 12: 1210–24. 10.1038/s41396-017-0037-1 29339824 PMC5932080

[37] Wu, Shengru , Lalle Hammarstedt‐Nordenvall , Mattias Jangard , Liqin Cheng , Sebastian Alexandru Radu , Pia Angelidou , et al. 2021. “Tonsillar Microbiota: A Cross‐Sectional Study of Patients with Chronic Tonsillitis or Tonsillar Hypertrophy.” mSystems 6: e01302–20. 10.1128/mSystems.01302-20 33688019 PMC8547005

[38] Liu, Juan , Li Cui , Xinmin Yan , Xinyuan Zhao , Jinluo Cheng , Lei Zhou , Jianbo Gao , et al. 2018. “Analysis of Oral Microbiota Revealed High Abundance of Prevotella Intermedia in Gout Patients.” Cellular Physiology and Biochemistry 49: 1804–12. 10.1159/000493626 30231244

[39] Li, Daxu , Weijun Xi , Zhe Zhang , Le Ren , Chunni Deng , Jianghao Chen , et al. 2020. “Oral Microbial Community Analysis of the Patients in the Progression of Liver Cancer.” Microbial Pathogenesis 149: 104479. 10.1016/j.micpath.2020.104479 32920149

[40] Chen, Wen‐Pei , Shih‐Hao Chang , Chuan‐Yi Tang , Ming‐Li Liou , Suh‐Jen Jane Tsai , and Yaw‐Ling Lin . 2018. “Composition Analysis and Feature Selection of the Oral Microbiota Associated with Periodontal Disease.” BioMed Research International 2018: 1–14. 10.1155/2018/3130607 PMC6276491 30581850

[41] Serra e Silva Filho, Wagner , Renato C. V. Casarin , Eduardo L. Nicolela Junior , Humberto M. Passos, Jr. , Antônio W. Sallum , and Reginaldo B. Gonçalves . 2014. “Microbial Diversity Similarities in Periodontal Pockets and Atheromatous Plaques of Cardiovascular Disease Patients.” PLoS One 9: e109761. 10.1371/journal.pone.0109761 25329160 PMC4199612

[42] Thukral, Rishi , Kirti Shrivastav , Vidhi Mathur , Animesh Barodiya , and Saurabh Shrivastav . 2017. “Actinomyces: A Deceptive Infection of Oral Cavity.” Journal of the Korean Association of Oral Maxillofacial Surgery 43: 282–5. 10.5125/jkaoms.2017.43.4.282 PMC5583205 28875145

[43] Stájer, Anette , Barrak Ibrahim , Márió Gajdács , Edit Urbán , and Zoltán Baráth . 2020. “Diagnosis and Management of Cervicofacial Actinomycosis: Lessons from Two Distinct Clinical Cases.” Antibiotics 9: 139. 10.3390/antibiotics9040139 32218154 PMC7235781

[44] Rosa, Rita , Maria La Giusy , Giuseppe Gattuso , Eugenio Pedullà , Ernesto Rapisarda , Daria Nicolosi , and Mario Salmeri . 2020. “Association of Oral Dysbiosis with Oral Cancer Development (Review).” Oncology Letters 19: 3045–58. 10.3892/ol.2020.11441 32211076 PMC7079586

[45] Wang, Yuan , Sa Wang , Chunyan Wu , Xi Chen , Zhuhui Duan , Qian Xu , Wen Jiang , et al. 2019. “Oral Microbiome Alterations Associated with Early Childhood Caries Highlight the Importance of Carbohydrate Metabolic Activities.” mSystems 4: e00450–19. 10.1128/mSystems.00450-19 31690590 PMC6832018

[46] Wang, Yao , Yao Zhang , Yun Qian , Yuan‐Hong Xie , Shan‐Shan Jiang , Zi‐Ran Kang , Ying‐Xuan Chen , Zhao‐Fei Chen , and Jing‐Yuan Fang . 2021. “Alterations in the Oral and Gut Microbiome of Colorectal Cancer Patients and Association With Host Clinical Factors.” International Journal of Cancer 149: 925–35. 10.1002/ijc.33596 33844851

[47] Wu, Juan , Shuo Xu , Chunjie Xiang , Qinhong Cao , Qiyi Li , Jiaqian Huang , Liyun Shi , Junfeng Zhang , and Zhen Zhan . 2018. “Tongue Coating Microbiota Community and Risk Effect on Gastric Cancer.” Journal of Cancer 9: 4039–48. 10.7150/jca.25280 30410609 PMC6218773

[48] Yang, Chia‐Yu , Yuan‐Ming Yeh , Hai‐Ying Yu , Chia‐Yin Chin , Chia‐Wei Hsu , Hsuan Liu , Po‐Jung Huang , et al. 2018. “Oral Microbiota Community Dynamics Associated with Oral Squamous Cell Carcinoma Staging.” Frontiers in Microbiology 9: 862. 10.3389/fmicb.2018.00862 29774014 PMC5943489

[49] Vogtmann, Emily , Yongli Han , J Gregory Caporaso , Nicholas Bokulich , Ashraf Mohamadkhani , Alireza Moayyedkazemi , Xing Hua , et al. 2020. “Oral Microbial Community Composition Is Associated With Pancreatic Cancer: A Case‐Control Study in Iran.” Cancer Medicine 9: 797–806. 10.1002/cam4.2660 31750624 PMC6970053

[50] Sun, Jing‐Hua , Xiao‐Lin Li , Jie Yin , Yi‐Hong Li , Ben‐Xiang Hou , and Zhongtao Zhang . 2018. “A Screening Method for Gastric Cancer By Oral Microbiome Detection.” Oncology Reports 39: 2217–24. 10.3892/or.2018.6286 29498406

[51] Zhao, Qiaofei , Tian Yang , Yifan Yan , Yu Zhang , Zhibin Li , Youchun Wang , Jing Yang , et al. 2020. “Alterations of Oral Microbiota in Chinese Patients with Esophageal Cancer.” Frontiers in Cellular and Infection Microbiology 10: 541144. 10.3389/fcimb.2020.541144 33194789 PMC7609410

[52] Chattopadhyay, Indranil , Mukesh Verma , and Madhusmita Panda . 2019. “Role of Oral Microbiome Signatures in Diagnosis and Prognosis of Oral Cancer.” Technology in Cancer Research & Treatment 18: 1533033819867354. 10.1177/1533033819867354 31370775 PMC6676258

[53] Yahara, Hiroko , Akimitsu Hiraki , Yutaka Maruoka , Aki Hirabayashi , Masato Suzuki , and Koji Yahara . 2020. “Shotgun Metagenome Sequencing Identification of a Set of Genes Encoded by Actinomyces Associated with Medication‐Related Osteonecrosis of the Jaw.” PLoS One 15: e0241676. 10.1371/journal.pone.0241676 33253207 PMC7703938

[54] Mougeot, Jean‐Luc C. , Micaela F. Beckman , Holden C. Langdon , Michael T. Brennan , and Farah Bahrani Mougeot . 2020. “Oral Microbiome Signatures in Hematological Cancers Reveal Predominance of Actinomyces and Rothia Species.” Journal of Clinical Medicine 9: 4068. 10.3390/jcm9124068 33348567 PMC7767039

[55] Li, Jun , Ying Li , Yu Zhou , Changzheng Wang , Benyan Wu , and Jun Wan . 2018. “Actinomyces and Alimentary Tract Diseases: A Review of Its Biological Functions and Pathology.” BioMed Research International 2018: e3820215. 10.1155/2018/3820215 PMC6129341 30225251

[56] Willis, Jesse R. , and Toni Gabaldón . 2020. “The Human Oral Microbiome in Health and Disease: From Sequences to Ecosystems.” Microorganisms 8: 308. 10.3390/microorganisms8020308 32102216 PMC7074908

[57] Graves, D. T. , J. D. Corrêa , and T. A. Silva . 2019. “The Oral Microbiota Is Modified by Systemic Diseases.” Journal of Dental Research 98: 148–56. 10.1177/0022034518805739 30359170 PMC6761737

[58] Lin, Wenyu , Qiaoyu Zhang , Yaojia Chen , Binhua Dong , Huifeng Xue , Huifang Lei , and Yanfang Lu , et al. 2022. “Changes of the Vaginal Microbiota in HPV Infection and Cervical Intraepithelial Neoplasia: A Cross‐Sectional Analysis.” Scientific Reports 12: 2812. 10.1038/s41598-022-06731-5 35181685 PMC8857277

[59] García‐García, Alejandra , Jaime Coronel‐Martínez , David Cantú‐de Leon , María Del Socorro Romero‐Figueroa , Yolanda Elizabeth Caballero‐Pantoja , Gauddy Lizeth Manzanares‐Leal , Miguel Rodriguez‐Morales , Horacio Sandoval‐Trujillo , and Ninfa Ramírez‐Durán . 2017. “Detection of Actinomyces Spp. in Cervical Exudates from Women with Cervical Intraepithelial Neoplasia or Cervical Cancer.” Journal of Medical Microbiology 66: 706–12. 10.1099/jmm.0.000485 28590243

[60] Du, Juan , Cecilia Nordfors , Andreas Ährlund‐Richter , Michal Sobkowiak , Mircea Romanitan , Anders Näsman , Sören Andersson , et al. 2012. “Prevalence of Oral Human Papillomavirus Infection among Youth, Sweden.” Emerging Infectious Diseases 18: 1468–71. 10.3201/eid1809.111731 22932445 PMC3437726

[61] Wu, Shengru , Luisa Warchavchik Hugerth , Ina Schuppe‐Koistinen , and Juan Du . 2022. “The Right Bug in the Right Place: Opportunities for Bacterial Vaginosis Treatment.” Npj Biofilms and Microbiomes 8: 34. 10.1038/s41522-022-00295-y 35501321 PMC9061781

[62] Hashioka, Sadayuki , Ken Inoue , Maiko Hayashida , Rei Wake , Arata Oh‐Nishi , and Tsuyoshi Miyaoka . 2018. “Implications of Systemic Inflammation and Periodontitis for Major Depression.” Frontiers in Neuroscience 12: 483. 10.3389/fnins.2018.00483 30072865 PMC6058051

[63] Kitamoto, Sho , Hiroko Nagao‐Kitamoto , Yizu Jiao , Merritt G. Gillilland , Atsushi Hayashi , Jin Imai , Kohei Sugihara , et al. 2020. “The Intermucosal Connection between the Mouth and Gut in Commensal Pathobiont‐Driven Colitis.” Cell 182: 447–62. 10.1016/j.cell.2020.05.048 32758418 PMC7414097

[64] Martínez, Isidoro , Juan C. Oliveros , Isabel Cuesta , Jorge de la Barrera , Vicente Ausina , Cristina Casals , et al. 2017. “Apoptosis, Toll‐Like, RIG‐I‐like and NOD‐like Receptors Are Pathways Jointly Induced by Diverse Respiratory Bacterial and Viral Pathogens.” Frontiers in Microbiology 8: 276. 10.3389/fmicb.2017.00276 28298903 PMC5331050

[65] Qi, Ying , Sheng‐Qi Zang , Juan Wei , Hong‐Chuan Yu , Zhao Yang , Hui‐Min Wu , Ying Kang , et al. 2021. “High‐Throughput Sequencing Provides Insights Into Oral Microbiota Dysbiosis in Association with Inflammatory Bowel Disease.” Genomics 113: 664–76. 10.1016/j.ygeno.2020.09.063 33010388

[66] Atarashi, Koji , Wataru Suda , Chengwei Luo , Takaaki Kawaguchi , Iori Motoo , Seiko Narushima , Yuya Kiguchi , et al. 2017. “Ectopic Colonization of Oral Bacteria in the Intestine Drives TH1 Cell Induction and Inflammation.” Science 358: 359–65. 10.1126/science.aan4526 29051379 PMC5682622

[67] Al Bataineh, Mohammad Tahseen , Nihar Ranjan Dash , Mohammed Elkhazendar , Dua'a Mohammad Hasan Alnusairat , Islam Mohammad Ismail Darwish , Mohamed Saleh Al‐Hajjaj , Qutayba Hamid , et al. 2020. “Revealing Oral Microbiota Composition and Functionality Associated with Heavy Cigarette Smoking.” Journal of Translational Medicine 18: 421. 10.1186/s12967-020-02579-3 33167991 PMC7653996

[68] Yang, Yaohua , Wei Zheng , Qiu‐Yin Cai , Martha J. Shrubsole , Zhiheng Pei , Robert Brucker , Mark D. Steinwandel , et al. 2019. “Cigarette Smoking and Oral Microbiota in Low‐Income and African‐American Populations.” Journal of Epidemiology and Community Health 73: 1108–15. 10.1136/jech-2019-212474 31563898 PMC6913090

[69] Yu, Guoqin , Stephen Phillips , Mitchell H. Gail , James J. Goedert , Michael S. Humphrys , Jacques Ravel , Yanfang Ren , and Neil E. Caporaso . 2017. “The Effect of Cigarette Smoking on the Oral and Nasal Microbiota.” Microbiome 5: 3. 10.1186/s40168-016-0226-6 28095925 PMC5240432

[70] Ganesan, Sukirth M. , Shareef M. Dabdoub , Haikady N. Nagaraja , Michelle L. Scott , Surya Pamulapati , Micah L. Berman , Peter G. Shields , Mary Ellen Wewers , and Purnima S. Kumar . 2020. “Adverse Effects of Electronic Cigarettes on the Disease‐Naive Oral Microbiome.” Science Advances 6: eaaz0108. 10.1126/sciadv.aaz0108 32518820 PMC7253170

[71] Lie, M. A. , G. A. Weijden , M. F. Timmerman , B. G. Loos , T. J. M. Steenbergen , and U. Velden . 1998. “Oral Microbiota In Smokers and Non‐Smokers in Natural and Experimentally‐Induced Gingivitis.” Journal of Clinical Periodontology 25: 677–86. 10.1111/j.1600-051x.1998.tb02505.x 9722273

[72] Odetto, Diego , Myriam Perrotta , Jose Martin Saadi , Carolina Beatriz Chacon , Pamela Ines Causa Andrieu , Alejandra Wernicke , and Marie Catherine Saez Perrotta . 2020. “Infection Versus Cancer: Management of Actinomyces Mimicking Cervical Cancer or Ovarian Cancer.” International Journal of Gynecologic Cancer 30: 1638–43. 10.1136/ijgc-2020-001800 PMC9040535 32753563

[73] Collins, Stuart , Terry P. Rollason , Lawrence S. Young , and Ciaran B. J. Woodman . 2010. “Cigarette Smoking is an Independent Risk Factor for Cervical Intraepithelial Neoplasia in Young Women: A Longitudinal Study.” European Journal of Cancer 46: 405–11. 10.1016/j.ejca.2009.09.015 19819687 PMC2808403

[74] Schwartz, Joel L. , Natalia Peña , Nadia Kawar , Andrew Zhang , Nicholas Callahan , Steven J. Robles , Andrew Griebel , and Guy R. Adami . 2021. “Old Age and Other Factors Associated with Salivary Microbiome Variation.” BMC Oral Health 21: 490. 10.1186/s12903-021-01828-1 34602059 PMC8489047

[75] Korona‐Glowniak, Izabela , Dominika Piatek , Emilia Fornal , Anna Lukowiak , Yuriy Gerasymchuk , Anna Kedziora , Gabriela Bugla‐Płoskonska , et al. 2021. “Patterns of Oral Microbiota in Patients with Apical Periodontitis.” Journal of Clinical Medicine 10: 2707. 10.3390/jcm10122707 34205290 PMC8234888

[76] Curtis, Mike A. , Patricia I. Diaz , and Thomas E. Van Dyke . 2020. “The Role of the Microbiota in Periodontal Disease.” Periodontol 2000 83: 14–25. 10.1111/prd.12296 32385883

[77] Douglas, Gavin M. , Vincent J. Maffei , Jesse R. Zaneveld , Svetlana N. Yurgel , James R. Brown , Christopher M. Taylor , Curtis Huttenhower , Morgan G. I. Langille , et al. 2020. “PICRUSt2 for Prediction of Metagenome Functions.” Nature Biotechnology 38: 685–8. 10.1038/s41587-020-0548-6 PMC7365738 32483366

[78] Du, Juan , Anders Näsman , Joseph W. Carlson , Torbjörn Ramqvist , and Tina Dalianis . 2011. “Prevalence of Human Papillomavirus (HPV) Types in Cervical Cancer 2003–2008 in Stockholm, Sweden, Before Public HPV Vaccination.” Acta Oncologica 50: 1215–9. 10.3109/0284186X.2011.584556 21726177

[79] Muñoz, Nubia , F Xavier Bosch , Silvia de Sanjosé , Rolando Herrero , Xavier Castellsagué , Keerti V. Shah , Peter J. F. Snijders , and Chris J. L. M. Meijer . 2003. “Epidemiologic Classification of Human Papillomavirus Types Associated with Cervical Cancer.” New England Journal of Medicine 348: 518–27. 10.1056/NEJMoa021641 12571259

[80] Mandal, Siddhartha , Will Van Treuren , Richard A. White , Merete Eggesbø , Rob Knight , and Shyamal D. Peddada . 2015. “Analysis of Composition of Microbiomes: A Novel Method for Studying Microbial Composition.” Microbial Ecology in Health & Disease 26: 27663. 10.3402/mehd.v26.27663 26028277 PMC4450248

[81] Hill‐Burns, Erin M. , Justine W. Debelius , James T. Morton , William T. Wissemann , Matthew R. Lewis , Zachary D. Wallen , Shyamal D. Peddada , et al. 2017. “Parkinson's Disease and Parkinson's Disease Medications Have Distinct Signatures of the Gut Microbiome: PD, Medications, and Gut Microbiome.” Movement Disorders 32: 739–49. 10.1002/mds.26942 28195358 PMC5469442

