# Supplementary material for: The salivary microbiota is altered in cervical dysplasia patients and influenced by conization
Source: Imeta. 2023 May 12;2(3):e108. doi: 10.1002/imt2.108 (PMC10989756; doi:10.1002/imt2.108)
Supplement: Supplementary file 1 — Supporting information. [file IMT2-2-e108-s002.docx]

**The salivary microbiota is altered in cervical dysplasia patients and influenced by conization**

**Running title: Salivary microbiota and cervical dysplasia**

Shengru Wu^1,2^, Liqin Cheng^1^, Alexandra A. L. Pennhag^1^, Maike Seifert^1^, Unnur Guðnadóttir^1^_,_ Lars Engstrand^1,3^, Miriam Mints^4^, †Sonia Andersson^4^, Juan Du^1,*^

**Author affiliations:**

1. Centre for Translational Microbiome Research, Department of Microbiology, Tumor and Cell Biology, Karolinska Institute, Stockholm, Sweden

2. College of Animal Science and Technology, Northwest A&F University, Yangling 712100, Shaanxi, China

3. Science for Life Laboratory, Karolinska Institute, Stockholm, Sweden

4. Department of Women’s and Children’s Health, Karolinska Institute, Stockholm, Sweden

† In memoriam of Dr. Sonia Andersson (1934-2022).

* Corresponding author: Juan Du

E-mail: juan.du@ki.se

**Supplementary files**

**Detail Materials and Methods**

**Figure S1.** Comparison of salivary microbial alpha diversity and beta diversity among all participants. (A) The Ace index was compared among participants with and without cervical dysplasia, and the control group. (B) The Ace index was compared among participants from the pre-dysplasia (+) and post-dysplasia (-) groups, and the control group. The Shannon (C) and Ace (D) indices were compared among participants from the control group and participants from the low-grade squamous intraepithelial lesion (LSIL), high-grade squamous intraepithelial lesion (HSIL), and within normal limits (WNL) groups. The Kruskal–Wallis test and Tukey-Kramer *post hoc* test were employed to test microbial alpha diversity differences between more than two groups. * FDR < 0.05, ** FDR < 0.01, *** FDR < 0.001. (E) The salivary microbial beta diversity (non-metric multidimensional scaling (NMDS) analysis with amplicon sequence variants (ASVs) based on Bray-Curtis distance matrices and ANOSIM analysis) was compared among participants from the control group and the LSIL, HSIL, and WNL groups.

**Figure S2.** Comparison of salivary microbial alpha and beta diversity among different age ranges of participants. The Shannon (A) and Ace (B) indices were compared among different age ranges of participants. The Kruskal–Wallis test and Tukey-Kramer *post hoc* test were employed to test microbial alpha diversity differences for more than two groups. ** FDR < 0.01, *** FDR < 0.001. (C) The salivary microbial beta diversity was compared among different age ranges of participants. The ANOSIM test was performed to compare salivary microbial beta diversity.

**Figure S3.** Comparison of salivary microbiota composition of all participants at the genus level. (A) The comparison of microbial composition among participants with and without cervical dysplasia, and the control group. (B) The comparison of microbial composition among participants from the pre-dysplasia (+) and post-dysplasia (-) groups, and the control group.

**Figure S4.** The microbial composition of all participants at the species level. (A) The microbial composition at the species level was compared between participants with and without cervical dysplasia, and the control group. (B) The microbial composition at the species level was compared among participants from the control group, and the LSIL, HSIL, and WNL groups. (C) The microbial composition at the species level was compared among patients from the pre-dysplasia (+) and post-dysplasia (-) groups, and the control group. Note: Only species with a relative abundance of more than 1% are listed.

**Figure S5.** The altered salivary microbiota genera among all participants in paired comparison. (A) The significantly changed salivary microbiota genera from the comparison between the participants with cervical dysplasia and the control group. (B) The significantly changed salivary microbiota genera from the comparison between the participants without cervical dysplasia and the control group. (C) The significantly changed salivary microbiota genera from the comparison between participants pre-dysplasia (+) and the control groups. (D) The significantly changed salivary microbiota genera from the comparison between participants post-dysplasia (-) and the control groups. The Mann-Whitney U test was carried out to compare the two groups. * FDR < 0.05, ** FDR < 0.01, *** FDR < 0.001. (E-G) The area under the curve (AUC) of receiver operating characteristic (ROC) analyses to identify the diagnostic accuracy of participants with cervical dysplasia from the healthy control group, based on host salivary *Haemophilus* (E), *Alloprevotella* (F), and *Prevotella* (G) abundance (*p* < 0.05).

**Figure S6.** Comparison of salivary microbial alpha diversity and beta diversity between participants with and without cervical dysplasia, and between participants pre-dysplasia (+) and post-dysplasia (-). (A) The Ace index was compared between participants with and without cervical dysplasia. (B) The Ace index was compared between participants pre-dysplasia (+) and post-dysplasia (-). The Mann-Whitney U test was carried out to compare the two groups. The Shannon (C) and Ace (D) indices were compared between the participants from the LSIL, HSIL, and WNL groups. The Kruskal–Wallis test with Tukey-Kramer *post hoc* test was employed to test microbial alpha diversity differences among more than two groups. (E) The salivary microbial beta diversity was compared among the participants from the LSIL, HSIL, and WNL groups. The ANOSIM analysis based on Bray-Curtis distance matrices were used to identify the beta diversity differences.

**Figure S7.** Comparison of salivary microbial alpha diversity, beta diversity, and the differential salivary genera between smokers and non-smokers. The Shannon index was compared between smoker and non-smoker groups with all participants (A), and with only vaginal examination participants (B). The Ace index was compared between smokers and non-smokers with all participants (C), and with only vaginal examination participants (D). The Mann-Whitney U test was carried out to compare the two groups. The salivary microbial beta diversity was compared between smoker and non-smoker groups with all participants (E) and with only vaginal examination participants (F). The ANOSIM analysis based on Bray-Curtis distance matrices was used to identify the beta diversity differences. (G) The distribution of different microbiota types between smoker and non-smoker groups with all participants. The significantly changed salivary microbiota genera between smokers and non-smokers with all participants (H) and with only vaginal examination participants (I). The Mann-Whitney U test was carried out for the two groups comparing. * FDR < 0.05, ** FDR < 0.01.

**Table S1.** The contribution of different variations to salivary microbial composition among all 67 participants was analyzed using permutational multivariate analysis of variance (PERMANOVA).

**Table S2.** The contribution of different variations to salivary microbial composition among the 60 participants from the control, pre-dysplasia (+), and post-dysplasia (-) groups was analyzed using PERMANOVA.

**Table S3.** The contribution of different variations to salivary microbial composition among the 47 vaginal examination participants was analyzed using PERMANOVA.

**Table S4.** The contribution of different variations to salivary microbial composition among the 40 vaginal examination participants from the pre-dysplasia (+) and post-dysplasia (-) groups was analyzed using PERMANOVA.

**Table S5.** Clinical characteristics of the participants who visited for vaginal examination.

**Table S6.** Questionnaire data of all the participants recruited in the study.

**Detail Materials and Methods**

**Study design, population, diagnosis**

This is a cross-sectional study involving 47 women who visited the Karolinska University Hospital for vaginal examination in Stockholm, Sweden. The enrolled women were examined according to national guidelines using cytology co-testing [1]. The liquid-based method (ThinPrep^®^, Hologic, USA) was used for cytology and the Cobas 4800 assay (Roche Molecular Diagnostics, USA) for standard high-risk HPV (HR-HPV) testing [2,3]. Briefly, samples were taken from the endocervix using cervical brushes and from the ectocervix with plastic spatulas. The samples were transferred into PreservCyt liquid-based cytology (LBC) vials according to European guidelines [1]. The LBC was carried out at the Cytology Department of the Karolinska University Hospital using the Bethesda system [4]. All participants were examined with colposcopy, performed by one of two gynecologists (Dr. Andersson or Dr. Mints). Punch biopsies were obtained from visible lesions and histologically graded by following the standard procedures at Karolinska University Hospital [5]. In addition, 20 healthy women volunteers who went for routine dental examination at a dental clinic in Stockholm, Sweden were recruited and provided saliva samples. No volunteer reported having a notable vaginal disease based on the questionnaires, and they were grouped as the control group. All participants had not taken antibiotics in the past month and had provided written informed consent to take part in the present study.

Out of 47 participants who visited hospital for vaginal examination, 8 had low‐grade squamous intraepithelial lesion (LSIL), 19 were high‐grade squamous intraepithelial lesion (HSIL), and 20 were within normal limits (WNL) according to histological analyses (Table S5). We grouped individuals with WNL status into the dysplasia negative (without dysplasia) group and LSIL combined with HSIL as the dysplasia positive (with dysplasia) group. Among the 28 participants who visit hospital for checking dysplasia, 24 of them were diagnosed with dysplasia and grouped into the pre-dysplasia (+) group. Among the 19 participants visited for a follow-up examination after conization treatment, 16 were dysplasia free and grouped as the post-dysplasia (-) group. Detailed diagnostic information on the 47 participants is listed in Table S5 and Fig. 1A. The participants were enrolled between October 2017 and October 2018, and the control group samples were collected during a similar period. Saliva samples were collected with SalivaGene Collector (Invitek Molecular GmbH, Germany) containing lyophilized DNA stabilization buffer. In total, 67 saliva samples were collected and frozen at -20°C until further DNA extraction. The study was approved by the Regional Ethical Board at Karolinska Institute, Stockholm, Sweden (ethical permission number 2017/725-31 and 2019-04201) and reported following the STORMS guideline [6].

**Q****uestionnaire scores**

During the first visiting, all the participants were asked to fill in the questionnaire about their lifestyle. The questions contain the basic information on e.g., age, place of birth, dentist appointment in the last 3 months, antibiotic use in the last 3 months, allergies, regular medicine, HPV vaccination, iron or vitamins intake, probiotic intake in the last few days, animal contact, traveling abroad during past 3 months, smoking habits, snuff habits, drinking habits, and eating habits (vegetarian or not, and fish, sweet drinks with sugar, sweet drinks without sugar, whole grain bread, fruit, vegetables/root vegetables intake) [7]. The participants were also grouped according to their responses on the lifestyle questionnaire in Table S6, which was then used for further permutational multivariate analysis of variance (PERMANOVA) to identify the main factors that correlate with salivary microbiota.

**DNA extraction**

The saliva samples were homogenized with ZR bashing beads (0.1 and 0.5 mm from Zymo Research, USA) for 1 min at 1,600 rpm with the 96 FastPrep machine (MP Biomedicals, USA). Then, 200 µl of the bead-beaten samples were incubated with 20 µl of lysozyme buffer (20 mM Tris-Cl, 2 mM sodium-EDTA, 100 g/ml lysozyme; Sigma, St. Louis, USA) for 60 min at 37°C, 1000 rpm. Samples were further digested with 20 µl proteinase K (20 mg/ml; Sigma, USA) for 30 min at 55°C, 250 rpm. The purification was performed with the Quick DNA Magbead Plus Kit (Zymo Research, USA) according to the manufacturer’s instruction and our previous study [8–10]. During the DNA extraction process, DNA/RNA Shield and ZymoBIOMICS Microbial Community Bacterial Standard (Zymo Research, USA) were used as the negative and positive extraction controls, respectively. The purified DNA was stored at -20°C until further microbiota sequencing.

**Oral HPV genotyping on participants for vaginal examination**

The extracted DNA was used for HPV genotyping with MAGPIX instrument according to our published papers [8,11–13]. Briefly, polymerase chain reaction (PCR) was carried out with broad-spectrum GP5+/6+ primers targeting the HPV L1 region, as well as HPV16 and HPV33 primers targeting the E6 region. The PCR product was further evaluated using a multiplex bead-based assay on a MAGPIX instrument (Luminex Inc., USA). In total, 15 oncogenic HPVs (HPV16, 18, 31, 33, 35, 39, 45, 51, 52, 56, 58, 59, 68, 73, 82), six probably oncogenic HPVs (HPV26, 30, 53, 66, 67, 69), and six non-oncogenic HPVs (HPV6, 11, 42, 43, 44, 70) were analyzed.

**Oral microbiota sequencing**

The V3–V4 regions of the 16S rRNA genes were amplified with Illumina sequencing index-binding primer pairs 341F/805R using a previously established automated pipeline [9]. For library preparation, DNA-free water and ZymoBIOMICS Microbial Community DNA Standard (Cat. No. D6305, Zymo Research, USA) were included as the negative and positive PCR controls. The negative and positive controls from the extraction phase were also submitted to library preparation and sequencing. Thereafter, paired end sequences (2 × 300 bp) of prepared libraries were generated on an Illumina MiSeq sequencing platform (Illumina, USA) with MiSeq Reagent Kit v3 (Illumina, USA).

**Bioinformatic analyses**

After demultiplexing, the sequences were merged with FLASH (v1.2.11) [14] and quality was filtered with fastp (0.19.6) [15]. The sequences that passed quality control were de-noised using DADA2 [16] in the QIIME2 [17] pipeline with single nucleotide resolution. Notably, the negative control and negative PCR control were used for contamination filtering by the Decontam program [18,19]. All of the species in the positive control samples (ZymoBIOMICS Microbial Community Bacterial Standard and ZymoBIOMICS Microbial Community DNA Standard; Zymo Research, USA) were detected at similar levels as the actual composition, which indicates trustworthy sequencing data and excluded in the following data analysis. To minimize the effects of sequencing depth on alpha and beta diversity measure, the number of sequences from each sample was rarefied to lowest reads number of detected samples. Taxonomic assignment of amplicon sequence variants (ASVs) was performed using the Naive bayes consensus taxonomy classifier [20] implemented in QIIME2 and the Human Oral Microbiome Database (eHOMD) (V15.2) (http:// <http://www.homd.org/>) [21].

The following analysis on alpha and beta diversity was performed on filtered data using “q2-diversity” commands in QIIME 2 [22]. Briefly, ASV richness estimates (Ace) and diversity indices (Shannon) were carried out for microbiota alpha diversity, and non-metric multi-dimensional scaling (NMDS) analysis based on Bray-Curtis dissimilarity were used for microbiota beta diversity analysis. In addition, PERMANOVA with questionnaire scores (Table S6) was performed to compare the contribution of each potential effector and their covariate to the salivary microbiota. Furthermore, Partitioning Around Medoids (PAM) clustering was performed based on the Jensen-Shannon divergence (JSD). The best clustering K number was calculated using the Calinski-Harabasz (CH) index and further used to distinguish the microbiota types [23]. When the CH index reached the highest value (CH=18.84), the best K number = 3 (indicating 3 clusters is the best cluster way in the present study) was obtained. Then the Between-class analysis (BCA, K ≥ 3) was used for visualization.

Network graphs were calculated based on the correlation of the abundance from all tested genera using the Python package NetworkX and visualized in Cytoscape (v3.4.0) [24]. PICRUSt2 analysis (https://github.com/picrust/picrust2) was applied to predict metagenome functions in the samples [25]. Immune function and cancer-related pathways were predicted using the Kyoto Encyclopedia of Genes and Genomes (KEGG) orthologs (KEGG hierarchical level 3) [26].

**Diagnostic accuracy**

The area under the curve (AUC) of the receiver operating characteristic (ROC) was calculated to analyze the sensitivity and specificity of the diagnostic power of the following categories: oral microbes, cytology combined with vaginal HPV testing, and cytology combined with vaginal HPV testing plus salivary microbes.

**References**

[1] Jordan, J, P Martin-Hirsch, M Arbyn, U Schenck, J-J Baldauf, D Da Silva, A Anttila, et al. 2009. "European guidelines for clinical management of abnormal cervical cytology, part 2." Cytopathology 20:5–16. https://doi.org/10.1111/j.1365-2303.2008.00636.x.

[2] Bentz, S, Joel. 2005. "Liquid-based cytology for cervical cancer screening." Expert Review of Molecular Diagnostics 5:857–71. https://doi.org/10.1586/14737159.5.6.857.

[3] Monsonego, Joseph, Michael G Hudgens, Laurent Zerat, Jean-Claude Zerat, Kari Syrjänen, Philippe Halfon, Fabrice Ruiz, et al. 2011. "Evaluation of oncogenic human papillomavirus RNA and DNA tests with liquid-based cytology in primary cervical cancer screening: the FASE study." International Journal of Cancer 129:691–701. https://doi.org/10.1002/ijc.25726.

[4] Solomon, Diane, Diane Davey, Robert Kurman, Ann Moriarty, Dennis O'Connor, Marianne Prey, Stephen Raab, et al. 2002. "The 2001 Bethesda System: terminology for reporting results of cervical cytology." JAMA 287:2114–9. https://doi.org/10.1001/jama.287.16.2114.

[5] Richart, RM. 1973. "Cervical intraepithelial neoplasia." Pathology Annual 8:301–28.

[6] Mirzayi, Chloe, Audrey Renson, Genomic Standards Consortium, Massive Analysis and Quality Control Society, Fatima Zohra, Shaimaa Elsafoury, Ludwig Geistlinger, et al. 2021. "Reporting guidelines for human microbiome research: the STORMS checklist." Nature Medicine 27:1885–92. https://doi.org/10.1038/s41591-021-01552-x.

[7] Bostanci, Nagihan, Maria Christine Krog, Luisa W Hugerth, Zahra Bashir, Emma Fransson, Fredrik Boulund, Georgios N Belibasakis, et al. 2021. "Dysbiosis of the Human Oral Microbiome During the Menstrual Cycle and Vulnerability to the External Exposures of Smoking and Dietary Sugar." Frontiers in Cellular and Infection Microbiology 11:625229. https://doi.org/10.3389/fcimb.2021.625229.

[8] Ährlund-Richter, Andreas, Liqin Cheng, Yue O O Hu, Mikaela Svensson, Alexandra A L Pennhag, Ramona G Ursu, Linnea Haeggblom, et al. 2019. "Changes in Cervical Human Papillomavirus (HPV) Prevalence at a Youth Clinic in Stockholm, Sweden, a Decade After the Introduction of the HPV Vaccine." Frontiers in Cellular Infection Microbiology 9:59. https://doi.org/10.3389/fcimb.2019.00059.

[9] Wu, Shengru, Lalle Hammarstedt-Nordenvall, Mattias Jangard, Liqin Cheng, Sebastian Alexandru Radu, Pia Angelidou, Yinghua Zha, et al. 2021. "Tonsillar Microbiota: a Cross-Sectional Study of Patients with Chronic Tonsillitis or Tonsillar Hypertrophy." mSystems 6:e01302-20. https://doi.org/10.1128/mSystems.01302-20.

[10] Hugerth, LW, Seifert M, Pennhag A a. L, Du J, Hamsten MC, Schuppe-Koistinen I, et al. 2018. "A comprehensive automated pipeline for human microbiome sampling, 16S rRNA gene sequencing and bioinformatics processing," Cold Spring Harbor Laboratory 2018:286526. https://doi.org/10.1101/286526.

[11] Cheng, Liqin, Johanna Norenhag, Yue O O Hu, Nele Brusselaers, Emma Fransson, Andreas Ährlund-Richter, Unnur Guðnadóttir, et al. 2020. "Vaginal microbiota and human papillomavirus infection among young Swedish women." Npj Biofilms and Microbiomes 6:1–10. https://doi.org/10.1038/s41522-020-00146-8.

[12] Du, Juan, Anders Näsman, Joseph W Carlson, Torbjörn Ramqvist, Tina Dalianis. 2011. "Prevalence of human papillomavirus (HPV) types in cervical cancer 2003-2008 in Stockholm, Sweden, before public HPV vaccination." Acta Oncologica 50:1215–9. https://doi.org/10.3109/0284186X.2011.584556.

[13] Muñoz, Nubia, F Xavier Bosch, Silvia de Sanjosé, Rolando Herrero, Xavier Castellsagué, Keerti V Shah, Peter J F Snijders, et al. 2003. "Epidemiologic classification of human papillomavirus types associated with cervical cancer." The New England Journal of Medicine 348:518–27. https://doi.org/10.1056/NEJMoa021641.

[14] Magoč, Tanja, Steven L Salzberg. 2011. "FLASH: fast length adjustment of short reads to improve genome assemblies." Bioinformatics 27:2957–63. https://doi.org/10.1093/bioinformatics/btr507.

[15] Chen, Shifu, Yanqing Zhou, Yaru Chen, Jia Gu. 2018. "fastp: an ultra-fast all-in-one FASTQ preprocessor." Bioinformatics 34:i884–90. https://doi.org/10.1093/bioinformatics/bty560.

[16] Callahan, J, Benjamin, Paul J McMurdie, Michael J Rosen, Andrew W Han, Amy Jo A Johnson, Susan P Holmes. 2016. "DADA2: High-resolution sample inference from Illumina amplicon data." Nature Methods 13:581–3. https://doi.org/10.1038/nmeth.3869.

[17] Bolyen, Evan, Jai Ram Rideout, Matthew R Dillon, Nicholas A Bokulich, Christian C Abnet, Gabriel A Al-Ghalith, Harriet Alexander, et al. 2019. "Reproducible, interactive, scalable and extensible microbiome data science using QIIME 2." Nature Biotechnology 37:852–7. https://doi.org/10.1038/s41587-019-0209-9.

[18] Davis, M, Nicole, Diana M Proctor, Susan P Holmes, David A Relman, Benjamin J Callahan. 2018. "Simple statistical identification and removal of contaminant sequences in marker-gene and metagenomics data." Microbiome 6:226. https://doi.org/10.1186/s40168-018-0605-2.

[19] Karstens, Lisa, Mark Asquith, Sean Davin, Damien Fair, W Thomas Gregory, Alan J Wolfe, Jonathan Braun, et al. 2019. "Controlling for Contaminants in Low-Biomass 16S rRNA Gene Sequencing Experiments." mSystems 4:e00290-19. https://doi.org/10.1128/mSystems.00290-19.

[20] Bokulich, A, Nicholas, Benjamin D Kaehler, Jai Ram Rideout, Matthew Dillon, Evan Bolyen, Rob Knight, Gavin A Huttley, et al. 2018. "Optimizing taxonomic classification of marker-gene amplicon sequences with QIIME 2’s q2-feature-classifier plugin." Microbiome 6:90. https://doi.org/10.1186/s40168-018-0470-z.

[21] Escapa, F, Isabel, Tsute Chen, Yanmei Huang, Prasad Gajare, Floyd E Dewhirst, Katherine P Lemon. 2018. "New Insights into Human Nostril Microbiome from the Expanded Human Oral Microbiome Database (eHOMD): a Resource for the Microbiome of the Human Aerodigestive Tract." mSystems 3:e00187-18. https://doi.org/10.1128/mSystems.00187-18.

[22] Estaki, Mehrbod, Lingjing Jiang, Nicholas A Bokulich, Daniel McDonald, Antonio González, Tomasz Kosciolek, Cameron Martino, et al. 2020. "QIIME 2 Enables Comprehensive End-to-End Analysis of Diverse Microbiome Data and Comparative Studies with Publicly Available Data." Current Protocols in Bioinformatics 70:e100. https://doi.org/10.1002/cpbi.100.

[23] Arumugam, Manimozhiyan, Jeroen Raes, Eric Pelletier, Denis Le Paslier, Takuji Yamada, Daniel R Mende, Gabriel R Fernandes, et al. 2011. "Enterotypes of the human gut microbiome." Nature 473:174–80. https://doi.org/10.1038/nature09944.

[24] Gomez, Andres, Josh L Espinoza, Derek M Harkins, Pamela Leong, Richard Saffery, Michelle Bockmann, Manolito Torralba, et al. 2017. "Host Genetic Control of the Oral Microbiome in Health and Disease." Cell Host Microbe 22:269-278.e3. https://doi.org/10.1016/j.chom.2017.08.013.

[25] Douglas, M, Gavin, Vincent J Maffei, Jesse R Zaneveld, Svetlana N Yurgel, James R Brown, Christopher M Taylor, Curtis Huttenhower, et al. 2020. "PICRUSt2 for prediction of metagenome functions." Nature Biotechnology 38:685–8. https://doi.org/10.1038/s41587-020-0548-6.

[26] Kanehisa, Minoru, Susumu Goto, Shuichi Kawashima, Yasushi Okuno, Masahiro Hattori. 2004. "The KEGG resource for deciphering the genome." Nucleic Acids Research 32:D277-280. https://doi.org/10.1093/nar/gkh063.

**Figure S1**


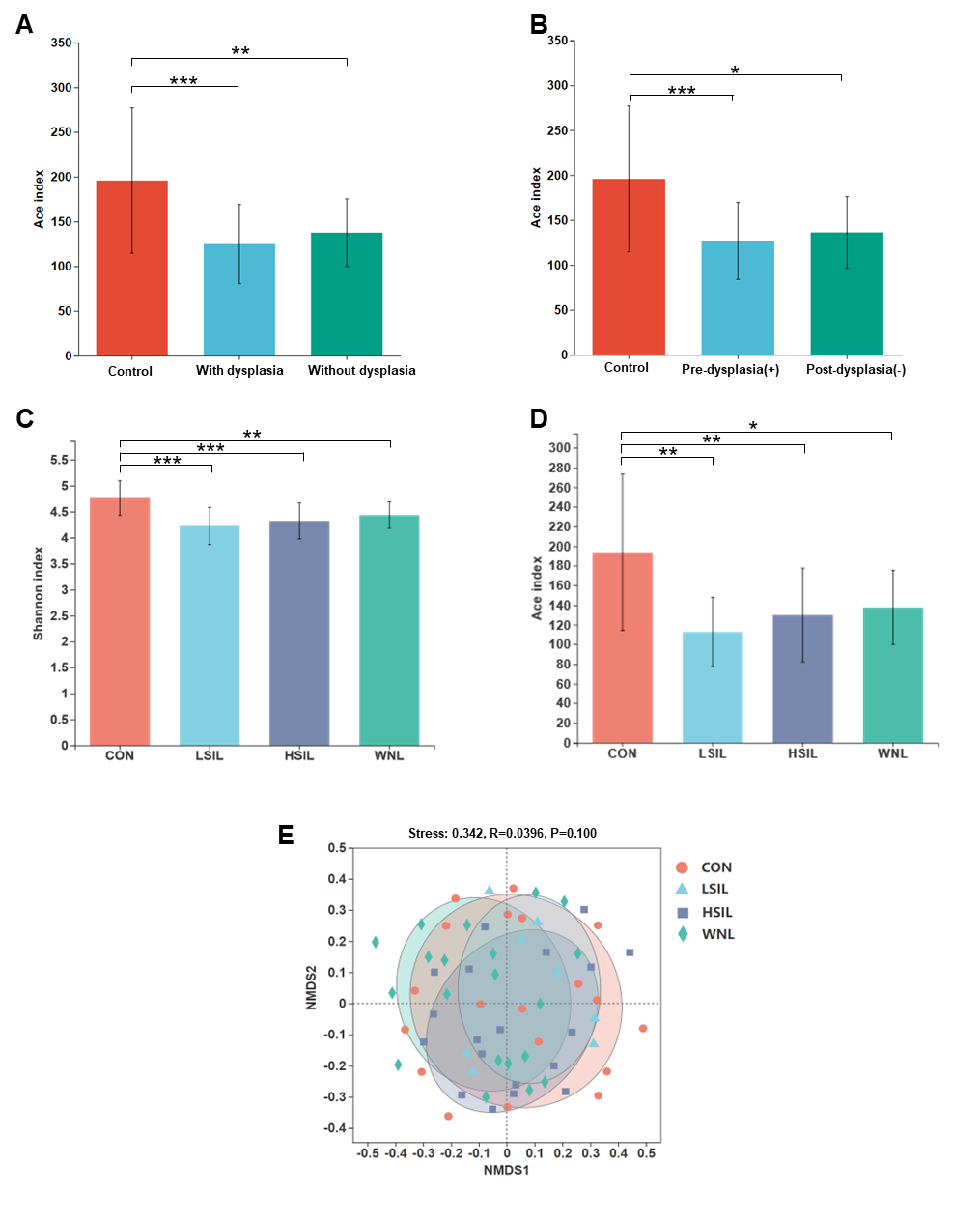


**Figure S2**


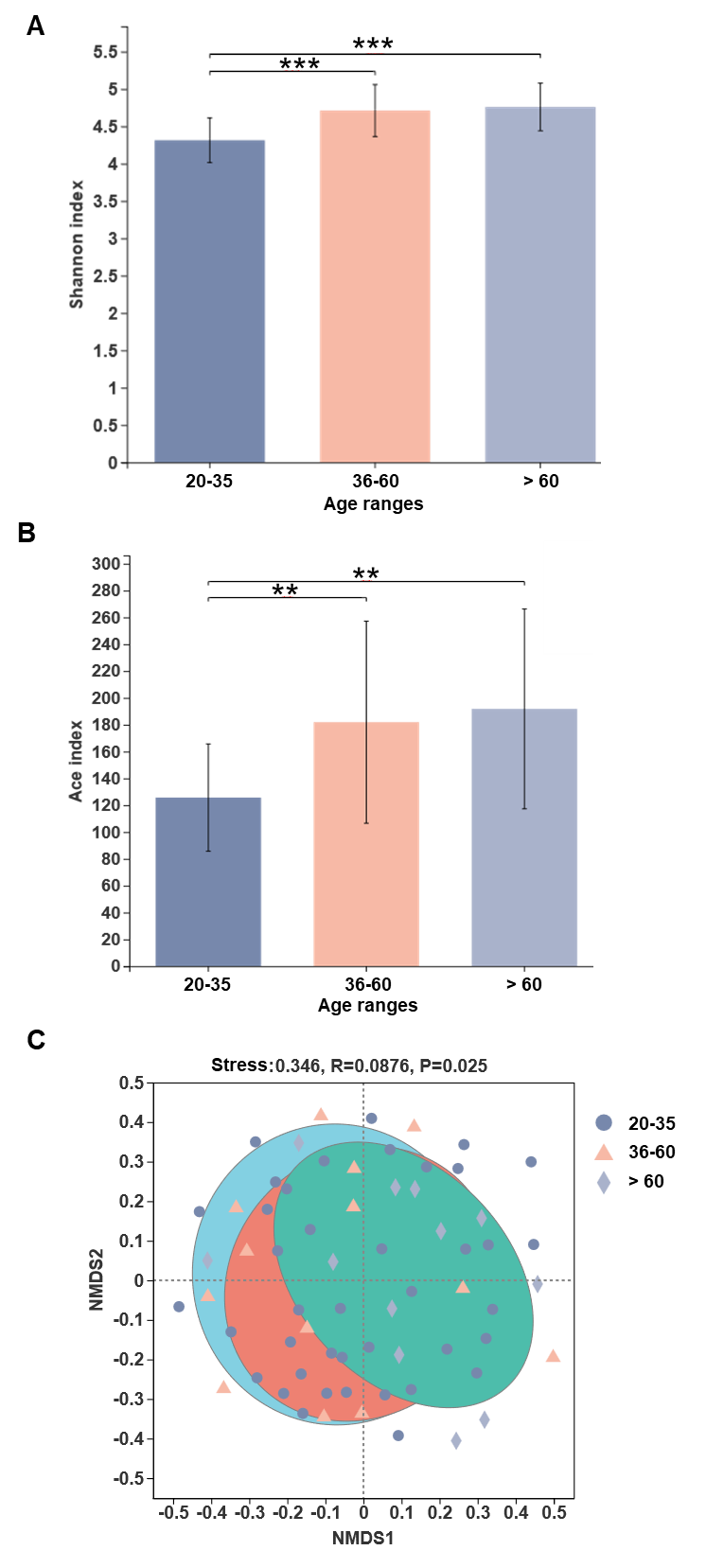


**Figure S3**


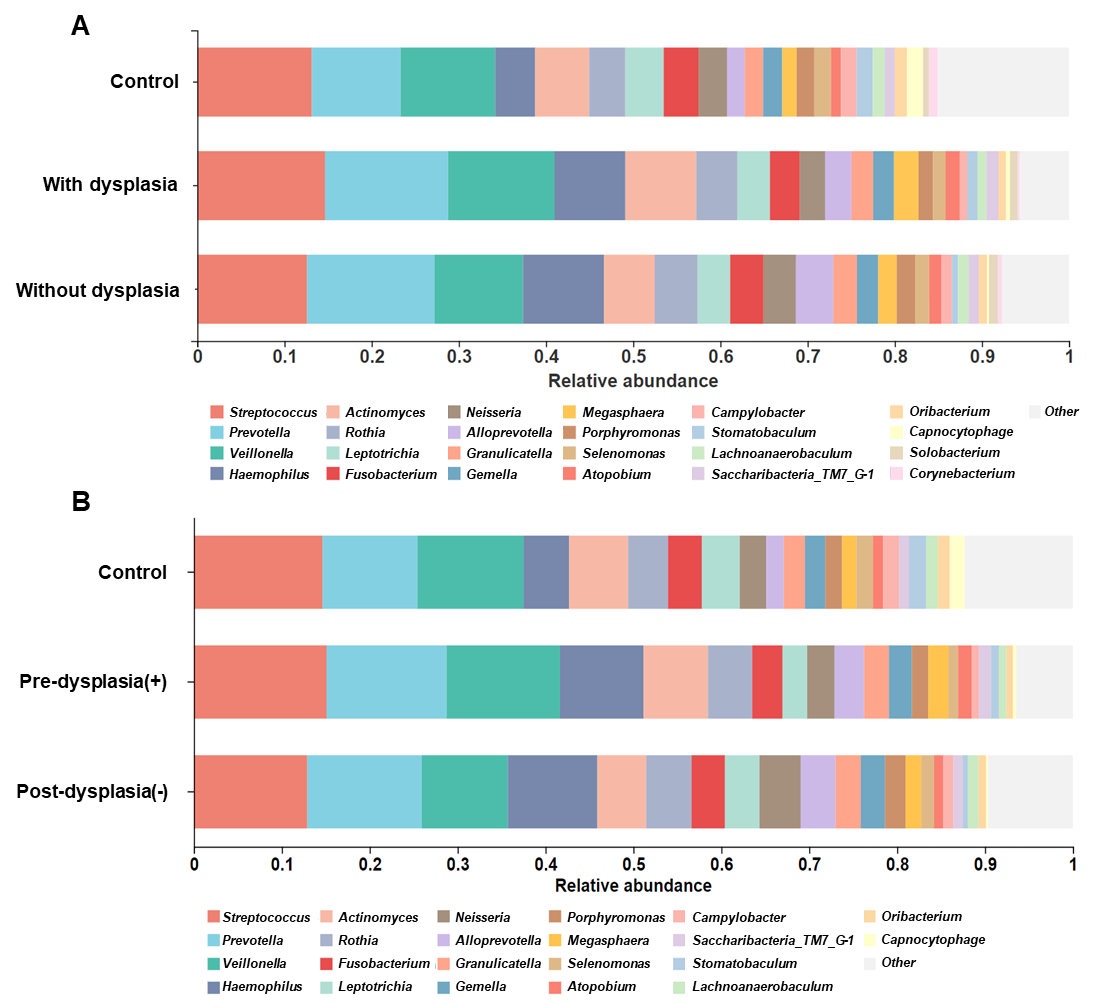


**Figure S4**


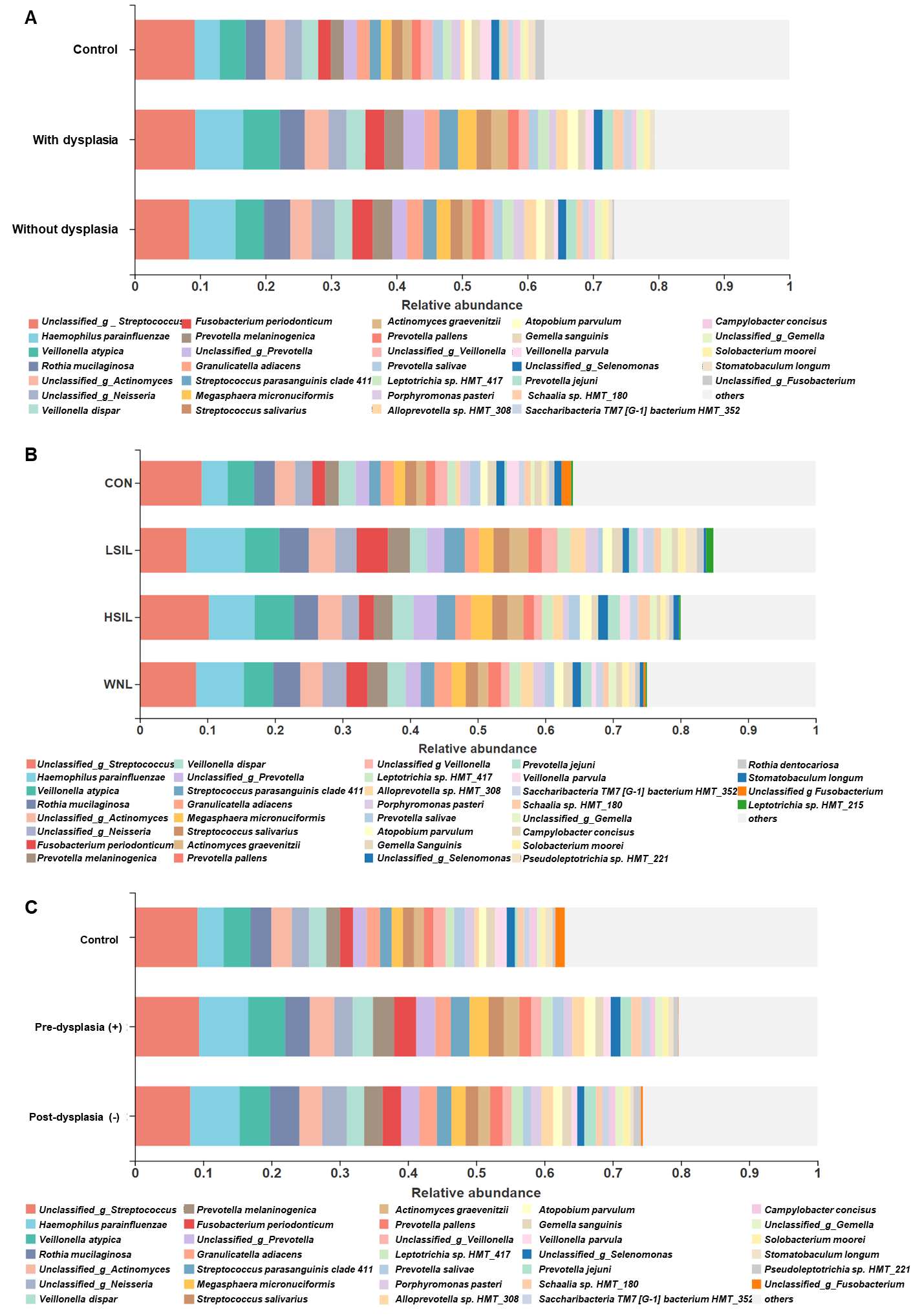


**Figure S5**


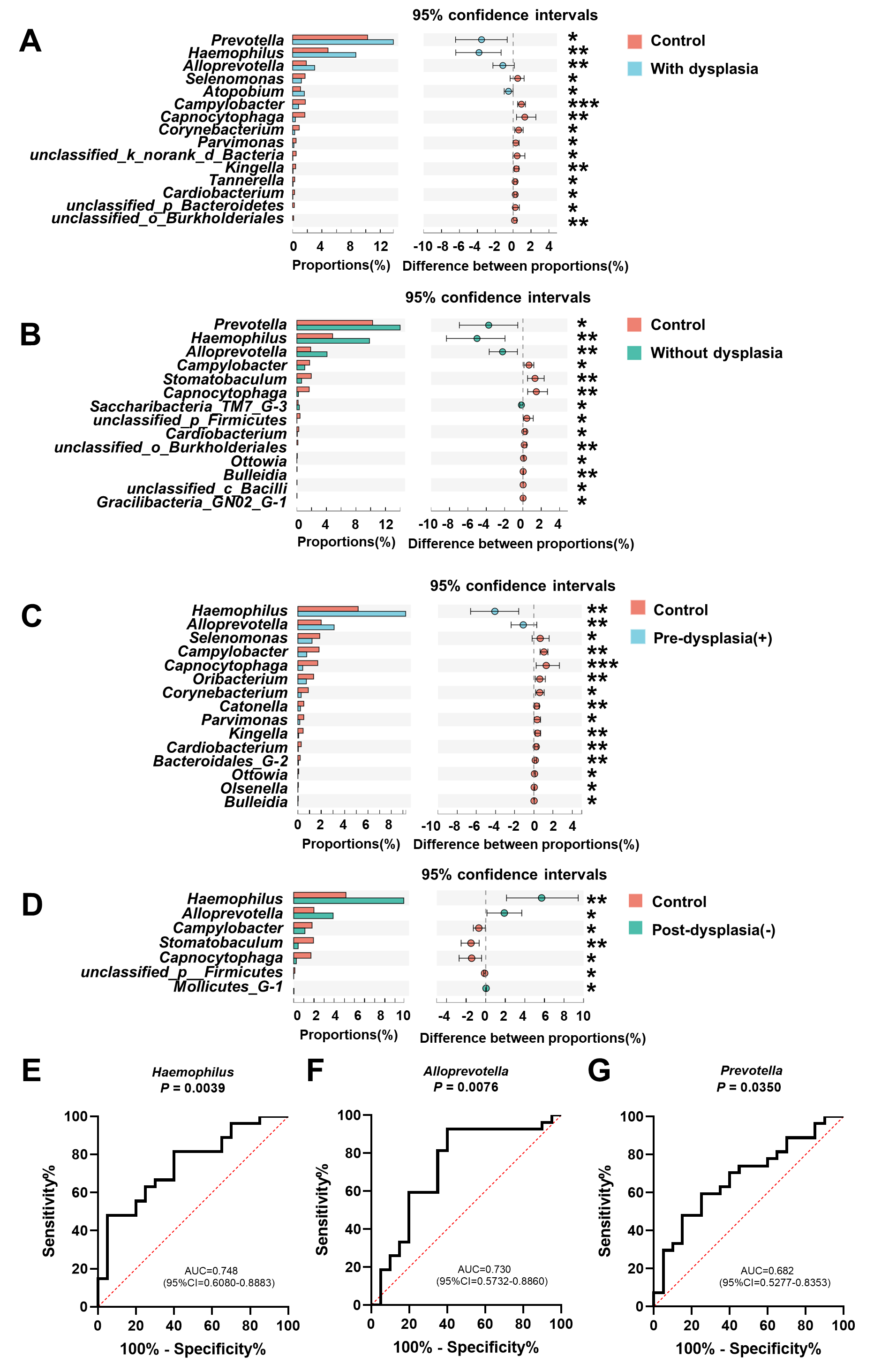


**Figure S6**


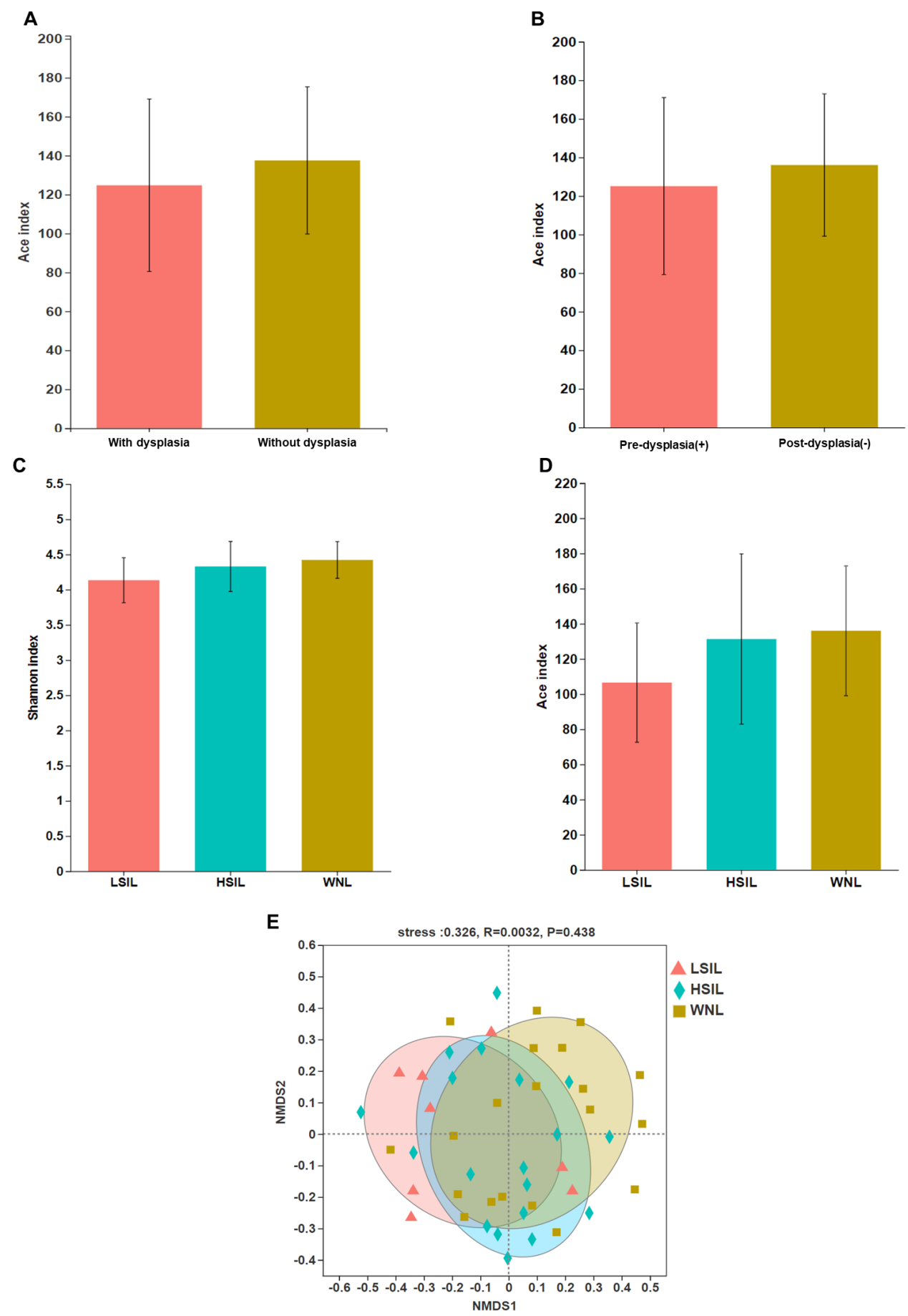


**Figure S7**


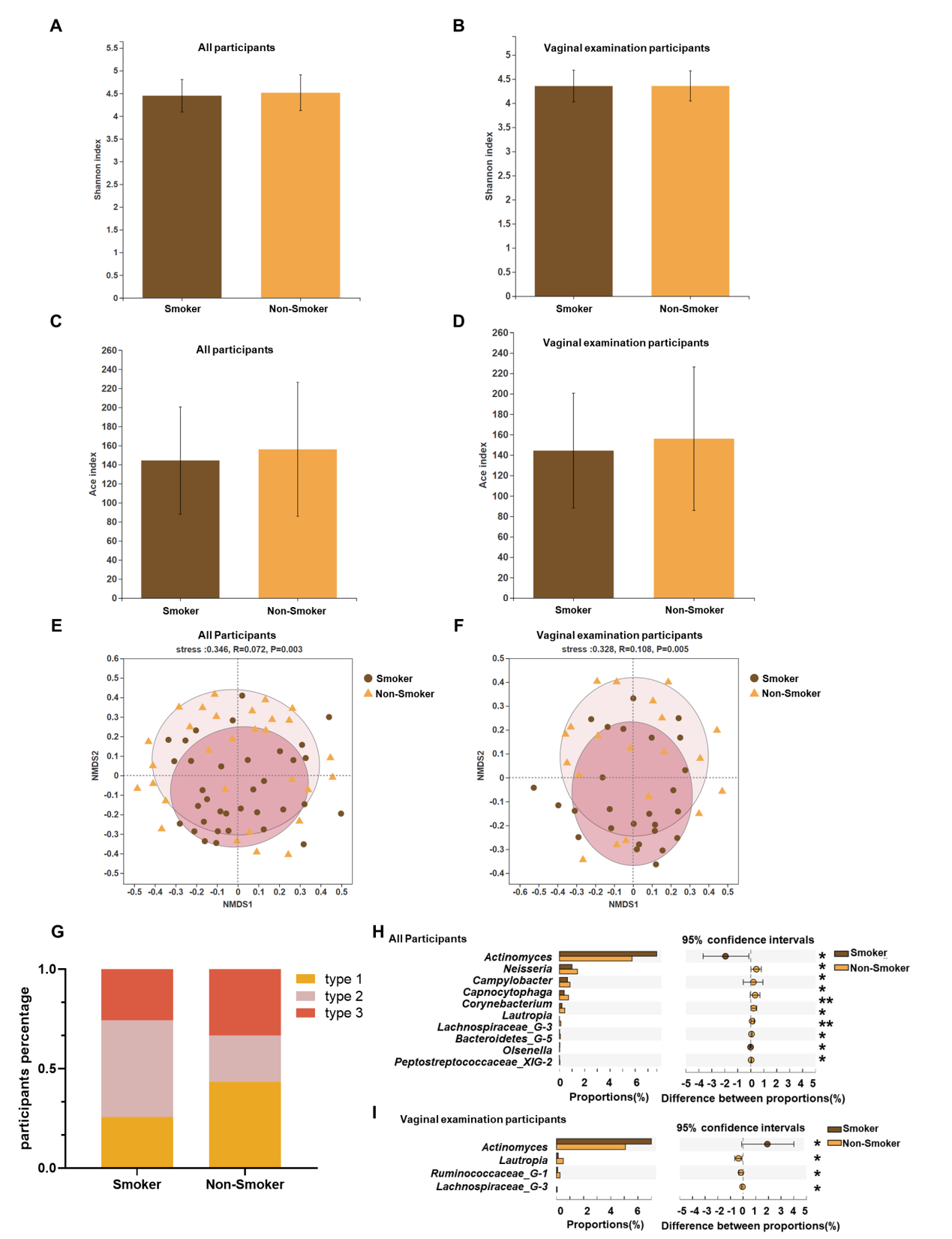


**Table S1. The contribution of different variations to salivary microbial composition among all 67 participants was analyzed using permutational multivariate analysis of variance (PERMANOVA).**

| Name | F.Models | R^2^ | Pr(>F) |
| --- | --- | --- | --- |
| Age | 1.218457 | 0.018806 | 0.002 |
| Allergies | 1.027199 | 0.015854 | 0.332 |
| Antibiotics | 1.055353 | 0.016288 | 0.156 |
| Been abroad | 1.017361 | 0.015702 | 0.331 |
| Dysplasia | 1.029403 | 0.031776 | 0.275 |
| Contact with animals | 1.016893 | 0.015695 | 0.353 |
| Dentist visit | 1.071273 | 0.016534 | 0.116 |
| Drinking | 1.023340 | 0.015794 | 0.344 |
| Fish | 0.947225 | 0.014620 | 0.84 |
| Fruit | 0.917570 | 0.014162 | 0.929 |
| HPV vaccination | 0.971815 | 0.014999 | 0.678 |
| Histology | 0.850129 | 0.013121 | 0.999 |
| Iron / Vitamins | 0.961835 | 0.014845 | 0.735 |
| Probiotics | 0.988846 | 0.015262 | 0.576 |
| Regular medicine | 0.930460 | 0.014361 | 0.897 |
| Smoke | 1.102780 | 0.017020 | 0.047 |
| Snuff | 0.912892 | 0.014090 | 0.961 |
| Sweet drinks sugar | 0.906840 | 0.013996 | 0.962 |
| Sweet drinks without sugar | 1.015272 | 0.015670 | 0.363 |
| Vegetables | 0.936104 | 0.014448 | 0.897 |
| Vegetarian | 0.891206 | 0.013755 | 0.98 |
| Whole grain bread | 0.970029 | 0.014972 | 0.69 |
| Residuals | - | 0.648231 | - |

**Table S2. The contribution of different variations to salivary microbial composition among the 60 participants from the control, pre-dysplasia (+), and post-dysplasia (-) groups was analyzed using PERMANOVA.**

| Name | F.Models | R^2^ | Pr(>F) |
| --- | --- | --- | --- |
| Age | 1.209914 | 0.021129 | 0.004 |
| Allergies | 1.065690 | 0.018610 | 0.140 |
| Antibiotics | 1.052828 | 0.018386 | 0.184 |
| Been abroad | 1.006806 | 0.017582 | 0.447 |
| Dysplasia with treatment | 1.039828 | 0.036317 | 0.173 |
| Contact with animals | 1.028294 | 0.017957 | 0.314 |
| Dentist visit | 1.058863 | 0.018491 | 0.164 |
| Drinking | 0.994993 | 0.017376 | 0.500 |
| Fish | 0.948409 | 0.016562 | 0.830 |
| Fruit | 0.927394 | 0.016195 | 0.895 |
| HPV vaccination | 0.930913 | 0.016257 | 0.894 |
| Histology | 0.993141 | 0.034686 | 0.528 |
| Iron / Vitamins | 1.008730 | 0.017615 | 0.424 |
| Probiotics | 1.039419 | 0.018151 | 0.254 |
| Regular medicine | 1.025770 | 0.017913 | 0.322 |
| Smoke | 1.110870 | 0.019399 | 0.037 |
| Snuff | 0.909751 | 0.015887 | 0.943 |
| Sweet drinks sugar | 0.990329 | 0.017294 | 0.558 |
| Sweet drinks without sugar | 0.973712 | 0.017004 | 0.686 |
| Vegetables | 0.951038 | 0.016608 | 0.794 |
| Vegetarian | 1.004190 | 0.017536 | 0.462 |
| Whole grain bread | 0.960130 | 0.016767 | 0.747 |
| Residuals | - | 0.576278 | - |

**Table S3. The contribution of different variations to salivary microbial composition among the 47 vaginal examination participants was analyzed using PERMANOVA.**

| Name | F.Models | R^2^ | Pr(>F) |
| --- | --- | --- | --- |
| Age | 0.993633 | 0.022432 | 0.509 |
| Allergies | 1.073968 | 0.024246 | 0.112 |
| Antibiotics | 0.994357 | 0.022448 | 0.537 |
| Been abroad | 0.967230 | 0.021836 | 0.674 |
| Dysplasia | 1.067566 | 0.024101 | 0.044 |
| Contact with animals | 0.959390 | 0.021659 | 0.729 |
| Dentist visit | 1.027029 | 0.023186 | 0.318 |
| Drinking | 1.015163 | 0.022918 | 0.389 |
| Fish | 0.963014 | 0.021741 | 0.749 |
| Fruit | 0.936663 | 0.021146 | 0.839 |
| HPV vaccination | 0.956728 | 0.021599 | 0.750 |
| Histology | 0.851101 | 0.019214 | 0.995 |
| Iron / Vitamins | 0.974511 | 0.022000 | 0.620 |
| Probiotics | 0.954381 | 0.021546 | 0.759 |
| Regular medicine | 0.910099 | 0.020546 | 0.937 |
| Smoke | 1.054831 | 0.023814 | 0.187 |
| Snuff | 0.909511 | 0.020533 | 0.919 |
| Sweet drinks sugar | 0.928830 | 0.020969 | 0.886 |
| Sweet drinks without sugar | 0.953278 | 0.021521 | 0.772 |
| Vegetables | 0.947377 | 0.021388 | 0.807 |
| Vegetarian | 0.886215 | 0.020007 | 0.974 |
| Whole grain bread | 0.970581 | 0.021912 | 0.686 |
| Residuals | - | 0.519241 | - |

**Table S4. The contribution of different variations to salivary microbial composition among the 40 vaginal examination participants from the pre-dysplasia (+) and post-dysplasia (-) groups was analyzed using PERMANOVA.**

| Name | F.Models | R^2^ | Pr(>F) |
| --- | --- | --- | --- |
| Age | 1.023674 | 0.026918 | 0.344 |
| Allergies | 1.113631 | 0.029284 | 0.067 |
| Antibiotics | 1.002697 | 0.026367 | 0.459 |
| Been abroad | 0.966029 | 0.025402 | 0.643 |
| Dysplasia with treatment | 1.186484 | 0.031200 | 0.008 |
| Contact with animals | 0.983646 | 0.025866 | 0.594 |
| Dentist visit | 1.015871 | 0.026713 | 0.386 |
| Drinking | 1.029431 | 0.027070 | 0.273 |
| Fish | 0.948312 | 0.024937 | 0.822 |
| Fruit | 0.942562 | 0.024785 | 0.791 |
| HPV vaccination | 0.942551 | 0.024785 | 0.832 |
| Histology | 0.908382 | 0.023887 | 0.923 |
| Iron / Vitamins | 1.010060 | 0.026560 | 0.416 |
| Probiotics | 1.029426 | 0.027070 | 0.302 |
| Regular medicine | 0.996524 | 0.026204 | 0.500 |
| Smoke | 1.066449 | 0.028043 | 0.157 |
| Snuff | 0.936565 | 0.024628 | 0.807 |
| Sweet drinks sugar | 0.994436 | 0.026149 | 0.519 |
| Sweet drinks without sugar | 0.955246 | 0.025119 | 0.742 |
| Vegetables | 0.969818 | 0.025502 | 0.654 |
| Vegetarian | 1.021077 | 0.026850 | 0.356 |
| Whole grain bread | 0.986066 | 0.025929 | 0.559 |
| Residuals | - | 0.420732 | - |

**Table S5. Clinical characteristics of the participants who visited for vaginal examination.**

| **Participants** | **Age** | **Dysplasia** | **Histology** | **Cytology** | **Treatment** | **Vaginal HPV** | **Oral HPV** |
| --- | --- | --- | --- | --- | --- | --- | --- |
| P1 | 22 | positive | LSIL | HSIL | post-treatment | positive | negative |
| P2 | 37 | negative | WNL | NILM | post-treatment | positive | negative |
| P3 | 32 | positive | HSIL | LSIL | post-treatment | positive | negative |
| P4 | 38 | negative | WNL | LSIL | pre-treatment | positive | negative |
| P5 | 37 | negative | WNL | NILM | post-treatment | negative | negative |
| P6 | unknown | negative | WNL | NILM | post-treatment | negative | negative |
| P7 | 48 | negative | WNL | NILM | post-treatment | negative | negative |
| P8 | 30 | negative | WNL | NILM | post-treatment | negative | negative |
| P9 | 24 | positive | HSIL | HSIL | pre-treatment | positive | negative |
| P10 | 28 | positive | LSIL | NILM | pre-treatment | positive | negative |
| P11 | 31 | negative | WNL | NILM | post-treatment | negative | negative |
| P12 | 26 | positive | HSIL | LSIL | pre-treatment | positive | negative |
| P13 | 24 | negative | WNL | NILM | post-treatment | negative | negative |
| P14 | 45 | negative | WNL | HSIL | post-treatment | positive | negative |
| P15 | 24 | positive | HSIL | HSIL | pre-treatment | positive | negative |
| P16 | 25 | negative | WNL | NILM | post-treatment | positive | negative |
| P17 | 27 | positive | HSIL | HSIL | pre-treatment | positive | negative |
| P18 | 30 | positive | HSIL | HSIL | pre-treatment | positive | negative |
| P19 | 33 | positive | HSIL | NILM | pre-treatment | positive | negative |
| P20 | 24 | negative | WNL | NILM | pre-treatment | positive | negative |
| P21 | 34 | positive | HSIL | HSIL | pre-treatment | positive | negative |
| P22 | 37 | negative | WNL | NILM | pre-treatment | positive | negative |
| P23 | 33 | positive | HSIL | HSIL | pre-treatment | positive | negative |
| P24 | 40 | negative | WNL | NILM | post-treatment | negative | negative |
| P25 | 31 | positive | LSIL | LSIL | pre-treatment | positive | negative |
| P26 | 64 | positive | HSIL | HSIL | pre-treatment | positive | negative |
| P27 | 32 | positive | LSIL | LSIL | post-treatment | positive | negative |
| P28 | 25 | positive | LSIL | LSIL | pre-treatment | positive | negative |
| P29 | 28 | positive | HSIL | HSIL | pre-treatment | positive | negative |
| P30 | 36 | positive | LSIL | HSIL | pre-treatment | positive | negative |
| P31 | 27 | positive | HSIL | HSIL | pre-treatment | positive | negative |
| P32 | 27 | positive | HSIL | HSIL | pre-treatment | positive | negative |
| P33 | 41 | positive | HSIL | HSIL | pre-treatment | positive | negative |
| P34 | 30 | negative | WNL | NILM | pre-treatment | negative | negative |
| P35 | 26 | negative | WNL | NILM | post-treatment | negative | negative |
| P36 | 34 | positive | HSIL | HSIL | pre-treatment | positive | negative |
| P37 | 27 | negative | WNL | NILM | post-treatment | negative | negative |
| P38 | 39 | negative | WNL | NILM | post-treatment | positive | negative |
| P39 | 28 | positive | LSIL | LSIL | pre-treatment | positive | negative |
| P40 | 32 | negative | WNL | NILM | post-treatment | positive | negative |
| P41 | 29 | negative | WNL | NILM | post-treatment | positive | negative |
| P42 | 45 | positive | LSIL | LSIL | pre-treatment | positive | negative |
| P43 | 29 | negative | WNL | NILM | post-treatment | negative | negative |
| P44 | 55 | positive | HSIL | HSIL | pre-treatment | positive | negative |
| P45 | 32 | positive | HSIL | HSIL | pre-treatment | positive | negative |
| P46 | 56 | positive | HSIL | HSIL | pre-treatment | positive | negative |
| P47 | 31 | positive | HSIL | HSIL | pre-treatment | positive | negative |

**Note:** For histological diagnoses, participants were classified as within normal limits (WNL), low-grade squamous intraepithelial lesion (LSIL), high-grade squamous intraepithelial lesion (HSIL). In the analysis, the LSIL and HSIL groups were identified as with dysplasia, and the WNL group was identified as without dysplasia.

For cytological diagnoses, participants were classified as negative for intraepithelial lesion or malignancy (NILM), low grade squamous intraepithelial lesion (LSIL), and high grade squamous intraepithelial lesion (HSIL).

For treatment, the 28 participants who came for investigation of potential dysplasia were grouped as pre-treatment, and the 20 participants who have been treated by conization and visited Karolinska University Hospital for follow-up examination were grouped as post-treatment.

**Table S6. Questionnaire data of all the participants recruited in the study.**

| **Sample** | **Age** | **Contact with animals** | **Been abroad during past 3 months** | **Antibiotics in the last 3 months** | **Dentist visit in the last 3 months** | **Smoking** | **Snuff** | **Drinking** | **Allergies** | **Regular medicine** |  | **HPV vaccination** | **Iron or Vitamins intakes** | **Probiotics in the last few days** | **Vegetarian** | **Fish** | **Sweet drinks with sugar** | **Sweet drinks without sugar** | **Whole grain bread** | **Fruit** | **Vegetable** |
| --- | --- | --- | --- | --- | --- | --- | --- | --- | --- | --- | --- | --- | --- | --- | --- | --- | --- | --- | --- | --- | --- |
| **CON1** | **77** | **No** | **No** | **No** | **No** | **Smoked** | **Never** | **Drunk** | **No** | **Yes** |  | **Unknown** | **Yes** | **No** | **No** | **Yes** | **frequently** | **frequently** | **rarely or never** | **frequently** | **frequently** |
| **CON2** | **72** | **Yes** | **No** | **No** | **No** | **Smoked** | **Never** | **Drunk** | **No** | **Yes** |  | **No** | **No** | **No** | **No** | **Yes** | **frequently** | **frequently** | **frequently** | **rarely or never** | **rarely or never** |
| **CON3** | **71** | **No** | **No** | **No** | **No** | **Smoked** | **Never** | **Never** | **No** | **Yes** |  | **No** | **No** | **No** | **No** | **Yes** | **frequently** | **frequently** | **frequently** | **rarely or never** | **frequently** |
| **CON4** | **73** | **No** | **Yes** | **No** | **No** | **Smoked** | **Never** | **Drunk** | **No** | **Yes** |  | **Unknown** | **No** | **No** | **No** | **Yes** | **frequently** | **frequently** | **frequently** | **rarely or never** | **rarely or never** |
| **CON5** | **71** | **No** | **Yes** | **No** | **No** | **Never** | **Never** | **Drunk** | **No** | **Yes** |  | **No** | **Yes** | **No** | **Yes** | **Yes** | **frequently** | **frequently** | **frequently** | **frequently** | **rarely or never** |
| **CON6** | **66** | **No** | **No** | **Yes** | **Yes** | **Smoked** | **Never** | **Drunk** | **No** | **Yes** |  | **No** | **Yes** | **Yes** | **No** | **Yes** | **frequently** | **frequently** | **frequently** | **frequently** | **frequently** |
| **CON7** | **70** | **No** | **No** | **No** | **No** | **Never** | **Never** | **Never** | **No** | **Yes** |  | **No** | **No** | **No** | **No** | **Yes** | **frequently** | **frequently** | **rarely or never** | **frequently** | **rarely or never** |
| **CON8** | **62** | **No** | **No** | **Yes** | **Yes** | **Smoked** | **Never** | **Drunk** | **No** | **Yes** |  | **Unknown** | **Yes** | **No** | **No** | **Yes** | **frequently** | **frequently** | **rarely or never** | **frequently** | **frequently** |
| **CON9** | **67** | **No** | **No** | **No** | **Yes** | **Never** | **Never** | **Drunk** | **No** | **Yes** |  | **Unknown** | **No** | **No** | **No** | **Yes** | **frequently** | **frequently** | **frequently** | **rarely or never** | **rarely or never** |
| **CON10** | **64** | **Yes** | **No** | **No** | **No** | **Never smoked** | **Never** | **Drunk** | **No** | **No** |  | **No** | **Yes** | **No** | **No** | **Yes** | **frequently** | **frequently** | **rarely or never** | **frequently** | **rarely or never** |
| **CON11** | **62** | **Yes** | **No** | **No** | **No** | **Smoked** | **Unknown** | **Drunk** | **No** | **No** |  | **No** | **No** | **No** | **No** | **Yes** | **frequently** | **frequently** | **frequently** | **frequently** | **rarely or never** |
| **CON12** | **77** | **No** | **No** | **No** | **No** | **Never** | **Never** | **Never** | **Yes** | **Yes** |  | **No** | **No** | **No** | **No** | **Yes** | **frequently** | **frequently** | **frequently** | **rarely or never** | **frequently** |
| **CON13** | **73** | **Yes** | **No** | **No** | **Yes** | **Smoked** | **Never** | **Drunk** | **No** | **Yes** |  | **No** | **Yes** | **No** | **No** | **Yes** | **frequently** | **frequently** | **rarely or never** | **rarely or never** | **rarely or never** |
| **CON14** | **79** | **Yes** | **No** | **No** | **Yes** | **Never** | **Never** | **Drunk** | **No** | **Yes** |  | **No** | **No** | **Yes** | **No** | **Yes** | **frequently** | **frequently** | **rarely or never** | **rarely or never** | **rarely or never** |
| **CON15** | **79** | **Yes** | **No** | **No** | **Yes** | **Smoked** | **Never** | **Drunk** | **No** | **Yes** |  | **Unknown** | **No** | **No** | **No** | **Yes** | **frequently** | **frequently** | **rarely or never** | **rarely or never** | **rarely or never** |
| **CON16** | **61** | **No** | **No** | **No** | **Yes** | **Never** | **Never** | **Drunk** | **Yes** | **Yes** |  | **No** | **No** | **No** | **No** | **Yes** | **frequently** | **frequently** | **rarely or never** | **rarely or never** | **rarely or never** |
| **CON17** | **78** | **Yes** | **No** | **No** | **Yes** | **Never** | **Never** | **Drunk** | **No** | **Yes** |  | **No** | **No** | **No** | **No** | **Yes** | **frequently** | **frequently** | **frequently** | **frequently** | **frequently** |
| **CON18** | **78** | **Yes** | **No** | **No** | **Yes** | **Never** | **Never** | **Drunk** | **Yes** | **Yes** |  | **Unknown** | **Yes** | **Yes** | **No** | **Yes** | **frequently** | **frequently** | **rarely or never** | **rarely or never** | **rarely or never** |
| **CON19** | **68** | **Yes** | **No** | **No** | **Yes** | **Never** | **Never** | **Drunk** | **No** | **Yes** |  | **No** | **Yes** | **Yes** | **No** | **Yes** | **frequently** | **rarely or never** | **rarely or never** | **rarely or never** | **rarely or never** |
| **CON20** | **62** | **Yes** | **No** | **No** | **Yes** | **Smoked** | **Never** | **Never** | **Yes** | **Yes** |  | **Unknown** | **Yes** | **No** | **No** | **Yes** | **frequently** | **rarely or never** | **frequently** | **rarely or never** | **rarely or never** |
| **P1** | **22** | **No** | **Yes** | **No** | **No** | **Smoked** | **Never** | **Drunk** | **Yes** | **No** |  | **Yes** | **No** | **No** | **Yes** | **Yes** | **rarely or never** | **rarely or never** | **rarely or never** | **frequently** | **frequently** |
| **P2** | **37** | **Yes** | **No** | **Yes** | **No** | **Never** | **Never** | **Drunk** | **Yes** | **Yes** |  | **No** | **Yes** | **No** | **Yes** | **Yes** | **frequently** | **rarely or never** | **frequently** | **frequently** | **frequently** |
| **P3** | **32** | **No** | **No** | **No** | **No** | **Smoked** | **Never** | **Drunk** | **No** | **No** |  | **No** | **No** | **No** | **No** | **Yes** | **rarely or never** | **rarely or never** | **frequently** | **frequently** | **frequently** |
| **P4** | **38** | **No** | **No** | **No** | **No** | **Never** | **Never** | **Never** | **No** | **Yes** |  | **Unknown** | **Yes** | **No** | **No** | **Yes** | **frequently** | **rarely or never** | **frequently** | **frequently** | **frequently** |
| **P5** | **37** | **No** | **No** | **No** | **No** | **Never** | **Never** | **Drunk** | **No** | **No** |  | **No** | **No** | **No** | **No** | **Yes** | **rarely or never** | **rarely or never** | **frequently** | **frequently** | **frequently** |
| **P6** | **Unknown** | **Unknown** | **Unknown** | **Unknown** | **Unknown** | **Unknown** | **Unknown** | **Unknown** | **Unknown** | **Unknown** |  | **Unknown** | **Unknown** | **Unknown** | **Unknown** | **Unknown** | **Unknown** | **Unknown** | **Unknown** | **Unknown** | **Unknown** |
| **P7** | **48** | **Yes** | **Yes** | **No** | **No** | **Never** | **Never** | **Drunk** | **No** | **No** |  | **Unknown** | **Yes** | **No** | **No** | **Yes** | **rarely or never** | **rarely or never** | **frequently** | **frequently** | **frequently** |
| **P8** | **30** | **No** | **Yes** | **No** | **No** | **Never** | **Never** | **Drunk** | **Yes** | **No** |  | **No** | **No** | **No** | **No** | **Yes** | **rarely or never** | **rarely or never** | **frequently** | **frequently** | **frequently** |
| **P9** | **24** | **Yes** | **No** | **No** | **No** | **Smoked** | **Snuff** | **Drunk** | **No** | **Yes** |  | **No** | **Yes** | **No** | **No** | **Yes** | **frequently** | **rarely or never** | **frequently** | **frequently** | **frequently** |
| **P10** | **28** | **No** | **Yes** | **No** | **No** | **Never** | **Never** | **Drunk** | **No** | **Yes** |  | **Yes** | **No** | **No** | **No** | **Yes** | **rarely or never** | **rarely or never** | **frequently** | **frequently** | **frequently** |
| **P11** | **31** | **No** | **Yes** | **No** | **No** | **Smoked** | **Snuff** | **Drunk** | **No** | **No** |  | **Yes** | **No** | **No** | **No** | **Yes** | **rarely or never** | **frequently** | **frequently** | **frequently** | **frequently** |
| **P12** | **26** | **No** | **Yes** | **No** | **No** | **Smoked** | **Never** | **Drunk** | **No** | **Yes** |  | **No** | **Yes** | **No** | **No** | **Yes** | **frequently** | **frequently** | **rarely or never** | **frequently** | **frequently** |
| **P13** | **24** | **Yes** | **No** | **No** | **Yes** | **Smoked** | **Never** | **Drunk** | **No** | **No** |  | **Unknown** | **No** | **No** | **No** | **Yes** | **frequently** | **frequently** | **frequently** | **frequently** | **frequently** |
| **P14** | **45** | **No** | **Yes** | **No** | **No** | **Never** | **Snuff** | **Drunk** | **No** | **Yes** |  | **No** | **No** | **No** | **No** | **Yes** | **frequently** | **rarely or never** | **frequently** | **frequently** | **frequently** |
| **P15** | **24** | **Yes** | **Yes** | **No** | **No** | **Never** | **Never** | **Drunk** | **No** | **No** |  | **No** | **No** | **No** | **No** | **Yes** | **frequently** | **frequently** | **frequently** | **frequently** | **frequently** |
| **P16** | **25** | **No** | **Yes** | **No** | **Yes** | **Never** | **Never** | **Drunk** | **No** | **No** |  | **Unknown** | **No** | **Yes** | **No** | **Yes** | **frequently** | **rarely or never** | **frequently** | **rarely or never** | **frequently** |
| **P17** | **27** | **Yes** | **No** | **No** | **No** | **Smoked** | **Snuff** | **Drunk** | **Yes** | **No** |  | **Yes** | **Yes** | **No** | **No** | **Yes** | **frequently** | **frequently** | **frequently** | **frequently** | **frequently** |
| **P18** | **30** | **Yes** | **Yes** | **No** | **No** | **Smoked** | **Snuff** | **Drunk** | **No** | **No** |  | **Yes** | **Yes** | **No** | **No** | **Yes** | **rarely or never** | **frequently** | **frequently** | **rarely or never** | **frequently** |
| **P19** | **33** | **No** | **No** | **No** | **No** | **Smoked** | **Never** | **Drunk** | **Yes** | **Yes** |  | **No** | **Yes** | **No** | **Yes** | **No** | **frequently** | **frequently** | **frequently** | **frequently** | **frequently** |
| **P20** | **24** | **Yes** | **Yes** | **No** | **No** | **Never** | **Never** | **Drunk** | **Yes** | **No** |  | **No** | **Yes** | **Yes** | **No** | **Yes** | **rarely or never** | **rarely or never** | **frequently** | **frequently** | **frequently** |
| **P21** | **34** | **Yes** | **No** | **No** | **No** | **Never** | **Never** | **Drunk** | **Yes** | **No** |  | **Yes** | **Yes** | **No** | **No** | **Yes** | **rarely or never** | **rarely or never** | **rarely or never** | **frequently** | **frequently** |
| **P22** | **37** | **No** | **Yes** | **No** | **No** | **Smoked** | **Snuff** | **Drunk** | **Yes** | **Yes** |  | **No** | **No** | **Unknown** | **No** | **Yes** | **rarely or never** | **rarely or never** | **frequently** | **frequently** | **frequently** |
| **P23** | **33** | **Yes** | **Yes** | **No** | **No** | **Never** | **Snuff** | **Drunk** | **No** | **No** |  | **No** | **Yes** | **No** | **No** | **Yes** | **frequently** | **rarely or never** | **frequently** | **frequently** | **frequently** |
| **P24** | **40** | **No** | **No** | **No** | **Yes** | **Never** | **Never** | **Drunk** | **Yes** | **No** |  | **No** | **No** | **No** | **No** | **Yes** | **rarely or never** | **frequently** | **frequently** | **frequently** | **frequently** |
| **P25** | **31** | **No** | **No** | **No** | **Yes** | **Smoked** | **Never** | **Drunk** | **No** | **No** |  | **Unknown** | **No** | **No** | **No** | **Yes** | **rarely or never** | **rarely or never** | **frequently** | **frequently** | **frequently** |
| **P26** | **64** | **No** | **No** | **No** | **Yes** | **Smoked** | **Never** | **Unknown** | **No** | **Yes** |  | **Yes** | **No** | **No** | **No** | **Yes** | **rarely or never** | **rarely or never** | **frequently** | **frequently** | **frequently** |
| **P27** | **32** | **No** | **Yes** | **No** | **No** | **Never** | **Never** | **Drunk** | **No** | **No** |  | **Unknown** | **No** | **No** | **No** | **Yes** | **rarely or never** | **rarely or never** | **frequently** | **frequently** | **frequently** |
| **P28** | **25** | **No** | **Yes** | **Yes** | **Yes** | **Smoked** | **Never** | **Drunk** | **No** | **No** |  | **No** | **Yes** | **No** | **No** | **Yes** | **rarely or never** | **rarely or never** | **frequently** | **frequently** | **frequently** |
| **P29** | **28** | **Yes** | **No** | **No** | **Yes** | **Smoked** | **Never** | **Drunk** | **No** | **No** |  | **No** | **No** | **No** | **No** | **Yes** | **frequently** | **frequently** | **frequently** | **frequently** | **frequently** |
| **P30** | **36** | **Yes** | **No** | **No** | **No** | **Never** | **Never** | **Never** | **No** | **Yes** |  | **No** | **Yes** | **No** | **Unknown** | **Unknown** | **frequently** | **rarely or never** | **frequently** | **frequently** | **frequently** |
| **P31** | **27** | **Yes** | **Yes** | **No** | **No** | **Smoked** | **Snuff** | **Drunk** | **No** | **Yes** |  | **No** | **Yes** | **No** | **Yes** | **No** | **frequently** | **frequently** | **rarely or never** | **frequently** | **frequently** |
| **P32** | **27** | **No** | **No** | **No** | **No** | **Never** | **Never** | **Drunk** | **No** | **No** |  | **No** | **No** | **No** | **No** | **Yes** | **frequently** | **rarely or never** | **frequently** | **frequently** | **frequently** |
| **P33** | **41** | **Yes** | **Yes** | **Yes** | **No** | **Smoked** | **Never** | **Drunk** | **Yes** | **Yes** |  | **Yes** | **Yes** | **No** | **No** | **Yes** | **frequently** | **rarely or never** | **frequently** | **frequently** | **frequently** |
| **P34** | **30** | **No** | **No** | **No** | **No** | **Never** | **Never** | **Drunk** | **Yes** | **Yes** |  | **No** | **Yes** | **No** | **No** | **Yes** | **rarely or never** | **rarely or never** | **frequently** | **frequently** | **frequently** |
| **P35** | **26** | **No** | **Yes** | **No** | **No** | **Smoked** | **Never** | **Drunk** | **No** | **No** |  | **No** | **No** | **No** | **No** | **Yes** | **frequently** | **frequently** | **rarely or never** | **frequently** | **frequently** |
| **P36** | **34** | **Yes** | **No** | **No** | **No** | **Smoked** | **Never** | **Drunk** | **Yes** | **Yes** |  | **Unknown** | **No** | **No** | **No** | **Yes** | **frequently** | **frequently** | **rarely or never** | **rarely or never** | **frequently** |
| **P37** | **27** | **No** | **Yes** | **No** | **No** | **Smoked** | **Never** | **Drunk** | **Yes** | **No** |  | **No** | **No** | **No** | **No** | **Yes** | **frequently** | **frequently** | **frequently** | **frequently** | **frequently** |
| **P38** | **39** | **No** | **Yes** | **No** | **No** | **Smoked** | **Never** | **Drunk** | **No** | **No** |  | **No** | **No** | **No** | **No** | **Yes** | **rarely or never** | **rarely or never** | **frequently** | **frequently** | **frequently** |
| **P39** | **28** | **Yes** | **Yes** | **No** | **No** | **Smoked** | **Never** | **Drunk** | **Yes** | **No** |  | **No** | **No** | **No** | **No** | **Yes** | **rarely or never** | **rarely or never** | **frequently** | **frequently** | **frequently** |
| **P40** | **32** | **No** | **Yes** | **Yes** | **Yes** | **Smoked** | **Never** | **Drunk** | **No** | **No** |  | **No** | **No** | **No** | **Yes** | **Yes** | **rarely or never** | **rarely or never** | **frequently** | **frequently** | **frequently** |
| **P41** | **29** | **Yes** | **Yes** | **No** | **No** | **Smoked** | **Never** | **Drunk** | **No** | **No** |  | **No** | **Yes** | **No** | **No** | **Yes** | **frequently** | **frequently** | **rarely or never** | **frequently** | **frequently** |
| **P42** | **45** | **No** | **Yes** | **No** | **No** | **Never** | **Never** | **Drunk** | **No** | **No** |  | **Unknown** | **No** | **No** | **No** | **Yes** | **rarely or never** | **rarely or never** | **frequently** | **frequently** | **frequently** |
| **P43** | **29** | **No** | **Yes** | **Yes** | **No** | **Smoked** | **Never** | **Drunk** | **No** | **No** |  | **No** | **No** | **No** | **No** | **Yes** | **frequently** | **rarely or never** | **rarely or never** | **frequently** | **frequently** |
| **P44** | **55** | **Yes** | **No** | **No** | **No** | **Smoked** | **Never** | **Drunk** | **Yes** | **Yes** |  | **No** | **Yes** | **No** | **No** | **Yes** | **rarely or never** | **frequently** | **frequently** | **frequently** | **frequently** |
| **P45** | **32** | **Yes** | **No** | **No** | **No** | **Never** | **Snuff** | **Drunk** | **Yes** | **Yes** |  | **No** | **Yes** | **No** | **No** | **Yes** | **rarely or never** | **frequently** | **frequently** | **frequently** | **frequently** |
| **P46** | **56** | **Yes** | **No** | **No** | **No** | **Smoked** | **Unknown** | **Drunk** | **No** | **Yes** |  | **No** | **Yes** | **No** | **No** | **Yes** | **rarely or never** | **rarely or never** | **frequently** | **frequently** | **frequently** |
| **P47** | **31** | **No** | **Yes** | **No** | **No** | **Never** | **Never** | **Drunk** | **Yes** | **No** |  | **No** | **Yes** | **No** | **No** | **Yes** | **rarely or never** | **frequently** | **rarely or never** | **rarely or never** | **frequently** |
