# Supplementary material for: The salivary microbiota is altered in cervical dysplasia patients and influenced by conization
Source: Imeta. 2023 May 12;2(3):e108. doi: 10.1002/imt2.108 (PMC10989756; doi:10.1002/imt2.108)
Supplement: Supplementary file 2 — Supporting information. [file IMT2-2-e108-s001.docx]

**Figure S1.** Comparison of salivary microbial alpha diversity and beta diversity among all participants. (A) The Ace index was compared among participants with and without cervical dysplasia, and the control group. (B) The Ace index was compared among patients from the pre-dysplasia (+) and post-dysplasia (-) groups, and the control group. The Shannon (C) and Ace (D) indices were compared among participants from the control group and participants from the low-grade squamous intraepithelial lesion (LSIL), high-grade squamous intraepithelial lesion (HSIL), and within normal limits (WNL) groups. The Kruskal–Wallis test and Tukey-Kramer *post hoc* test were employed to test microbial alpha diversity differences between more than two groups. * FDR▒<▒0.05, ** FDR▒<▒0.01, *** FDR▒<▒0.001. (E) The salivary microbial beta diversity (non-metric multidimensional scaling (NMDS) analysis with amplicon sequence variants (ASVs) based on Bray-Curtis distance matrices and ANOSIM analysis) was compared among participants from the control group and the LSIL, HSIL, and WNL groups.

**Figure S2.** Comparison of salivary microbial alpha and beta diversity among different age ranges of participants. The Shannon (A) and Ace (B) indices were compared among different age ranges of participants. The Kruskal–Wallis test and Tukey-Kramer *post hoc* test were employed to test microbial alpha diversity differences for more than two groups. * FDR▒<▒0.05, ** FDR▒<▒0.01, *** FDR▒<▒0.001. (C) The salivary microbial beta diversity was compared among different age ranges of participants. The ANOSIM test was performed to compare salivary microbial beta diversity.

**Figure S3.** Comparison of salivary microbiota composition of all participants at the genus level. (A) The comparison of microbial composition among participants with and without cervical dysplasia, and the control group. (B) The comparison of microbial composition among participants from the pre-dysplasia (+) and post-dysplasia (-) groups, and the control group.

**Figure S4.** The microbial composition of all participants at the species level. (A) The microbial composition at the species level was compared between participants with and without cervical dysplasia, and the control group. (B) The microbial composition at the species level was compared among participants from the control group, and the WNL, LSIL, HSIL groups. (C) The microbial composition at the species level was compared among patients from the pre-dysplasia (+) and post-dysplasia (-) groups, and the control group. Note: Only species with a relative abundance of more than 1% are listed.

**Figure S5.** The altered salivary microbiota genera among all participants in paired comparison. (A) The significantly changed salivary microbiota genera from the comparison between the participants with cervical dysplasia and the control group. (B) The significantly changed salivary microbiota genera from the comparison between the participants without cervical dysplasia and the control group. (C) The significantly changed salivary microbiota genera from the comparison between participants pre-dysplasia (+) and the control groups. (D) The significantly changed salivary microbiota genera from the comparison between participants post-dysplasia (-) and the control groups. The Mann-Whitney U test was carried out to compare the two groups. * FDR▒<▒0.05, ** FDR▒<▒0.01, *** FDR▒<▒0.001. (E-G) The area under the curve (AUC) of receiver operating characteristic (ROC) analyses helps to identify the diagnostic accuracy of participants with cervical dysplasia and healthy control group, based on host salivary *Haemophilus* (E), *Alloprevotella* (F), and *Prevotella* (G) abundance (*p*▒<▒0.05).

**Figure S6.** Comparison of salivary microbial alpha diversity and beta diversity between participants with and without cervical dysplasia, and between participants pre-dysplasia (+) and post-dysplasia (-). (A) The Ace index was compared between participants with and without cervical dysplasia. (B) The Ace index was compared between participants pre-dysplasia (+) and post-dysplasia (-). The Mann-Whitney U test was carried out to compare the two groups. The Shannon (C) and Ace (D) indices were compared between the participants from the WNL, LSIL, and HSIL groups. The Kruskal–Wallis test with Tukey-Kramer *post hoc* test was employed to test microbial alpha diversity differences among more than two groups. (E) The salivary microbial beta diversity was compared among the participants from the WNL, LSIL, and HSIL groups. The ANOSIM analysis based on Bray-Curtis distance matrices were used to identify the beta diversity differences.

**Figure S7.** Comparison of salivary microbial alpha diversity, beta diversity, and the differential salivary genera between smokers and non-smokers. The Shannon index was compared between smoker and non-smoker groups with all participants (A), and with only vaginal examination participants (B). The Ace index was compared between smokers and non-smokers with all participants (C), and with only vaginal examination participants (D). The Mann-Whitney U test was carried out to compare the two groups. The salivary microbial beta diversity was compared between smoker and non-smoker groups with all participants (E) and with only vaginal examination participants (F). The ANOSIM analysis based on Bray-Curtis distance matrices was used to identify the beta diversity differences. (G) The distribution of different microbiota types between smoker and non-smoker groups with all participants. The significantly changed salivary microbiota genera between smokers and non-smokers with all participants (H) and with only vaginal examination participants (I). The Mann-Whitney U test was carried out for the two groups comparing. * FDR▒<▒0.05, ** FDR▒<▒0.01.
